# Machine learning-based pathomics signature of histology slides as a novel prognostic indicator in primary central nervous system lymphoma

**DOI:** 10.1007/s11060-024-04665-8

**Published:** 2024-04-01

**Authors:** Ling Duan, Yongqi He, Wenhui Guo, Yanru Du, Shuo Yin, Shoubo Yang, Gehong Dong, Wenbin Li, Feng Chen

**Affiliations:** 1 Department of Neuro-Oncology, Cancer Center, Beijing Tiantan Hospital, Capital Medical University, No.119 West Nansihuan Road, Beijing, 100070 China; 2 Department of Pathology, Beijing Tiantan Hospital, Capital Medical University, No.119 West Nansihuan Road, Beijing, 100070 China

**Keywords:** Primary central nervous system lymphoma, Histopathological images, Pathomics, Prognosis

## Abstract

**Purpose:**

To develop and validate a pathomics signature for predicting the outcomes of Primary Central Nervous System Lymphoma (PCNSL).

**Methods:**

In this study, 132 whole-slide images (WSIs) of 114 patients with PCNSL were enrolled. Quantitative features of hematoxylin and eosin (H&E) stained slides were extracted using CellProfiler. A pathomics signature was established and validated. Cox regression analysis, receiver operating characteristic (ROC) curves, Calibration, decision curve analysis (DCA), and net reclassification improvement (NRI) were performed to assess the significance and performance.

**Results:**

In total, 802 features were extracted using a fully automated pipeline. Six machine-learning classifiers demonstrated high accuracy in distinguishing malignant neoplasms. The pathomics signature remained a significant factor of overall survival (OS) and progression-free survival (PFS) in the training cohort (OS: HR 7.423, *p* < 0.001; PFS: HR 2.143, *p* = 0.022) and independent validation cohort (OS: HR 4.204, *p* = 0.017; PFS: HR 3.243, *p* = 0.005). A significantly lower response rate to initial treatment was found in high Path-score group (19/35, 54.29%) as compared to patients in the low Path-score group (16/70, 22.86%; *p* < 0.001). The DCA and NRI analyses confirmed that the nomogram showed incremental performance compared with existing models. The ROC curve demonstrated a relatively sensitive and specific profile for the nomogram (1-, 2-, and 3-year AUC = 0.862, 0.932, and 0.927, respectively).

**Conclusion:**

As a novel, non-invasive, and convenient approach, the newly developed pathomics signature is a powerful predictor of OS and PFS in PCNSL and might be a potential predictive indicator for therapeutic response.

**Supplementary Information:**

The online version contains supplementary material available at 10.1007/s11060-024-04665-8.

## Introduction

Primary central nervous system lymphoma (PCNSL) is a rare and highly aggressive type of extranodal non-Hodgkin lymphoma that exclusively affects the brain, spinal cord, leptomeninges, and/or eyes. PCNSL comprises only ~ 4% of newly diagnosed central nervous system (CNS) tumors and 4–6% of all extranodal lymphomas in immunocompetent patients [1]. The incidence of PCNSL is between 0.3 and 0.6 cases per 1000000 people annually in the United States and has increased over the past four decades, particularly in patients older than 60 years [2–4]. Approximately 95% of PCNSLs are classified as diffuse large B-cell lymphoma (DLBCL), with a predominantly nongerminal center B-cell-like (non-GCB) immunophenotype [5]. Typical histopathological characteristics include a perivascular arrangement of highly proliferating tumor cells forming a unique angiocentric growth pattern [6]. Despite remarkable therapeutic progress, 15–25% of patients do not respond to chemotherapy, and 25–50% relapse after the initial response [7–9], resulting in a poor overall outcome. The 5-year overall survival (OS) rate ranges from 22.3% to 35% [10–12].

Currently, two prognostic models are commonly used to predict clinical outcomes in patients. One was developed by the International Extranodal Lymphoma Study Group (IELSG) and includes age, Performance Status (PS), serum lactate dehydrogenase (LDH), cerebrospinal fluid (CSF) protein, and deep brain involvement [13]. The other was developed by researchers at the Memorial Sloan-Kettering Cancer Center (MSKCC), which includes age and PS [14]. However, the levels of LDH or CSF protein (contraindications for lumbar puncture) at diagnosis are not always clear, which adds many limitations to the application of IELSG in clinical practice. The MSKCC model did not identify significant survival differences in several recent studies [15, 16]. New insights into the pathobiology of the disease and improved treatment approaches continue to challenge the application of these models. Thus, there is an urgent need to discover new biomarkers associated with prognosis.

Although convolutional pathological diagnosis is time-consuming, labor-intensive, and relies heavily on the pathologist’s subjective judgment, the evaluation of histological slides remains the gold standard for tumor diagnosis and staging. Computational pathology leverages advanced techniques to analyze large-scale pathological data, including histopathological images, genomic data, and clinical information, and has been shown to improve the efficiency, accuracy, and consistency of histopathological evaluations. An increasing number of histopathological analysis algorithms have been developed for tumor grading [17], automatic classification [18], and identification of lymph node metastases [19]. The Least Absolute Shrinkage and Selection Operator (LASSO) regression is an efficient machine learning method, which analyzes relationships between high-dimensional features and outcomes. It can minimize the potential collinearity of variables and has been reported to be valuable in the survival prediction of PCNSL [20, 21]. In addition, several recent studies have used digital pathology images to address survival prediction in various malignancies, including lung cancer [22], gastric cancer [23], gliomas [24], and lung metastasis in colorectal cancer [25]. Therefore, we aimed to explore the possibility of analyzing the automatic digital pathological features extracted from Hematoxylin and Eosin (H&E)-stained slides to predict prognosis in patients with PCNSL.

Here, in this study, we performed a fully automated pipeline to extract quantitative features, showed their ability to distinguish malignant neoplasms, developed and validated a novel pathomics score (Path-score) based on these features using the LASSO-Cox regression model in PCNSL patients. Furthermore, the Path-score has been proven to have an essential correlation with initial treatment response. Finally, we constructed a nomogram that combined the Path-score and clinical characteristics to conveniently predict patient outcomes and demonstrated better performance than existing prognostic models.

## Materials and methods

### Data cohort and study design

**Fig. 1:**
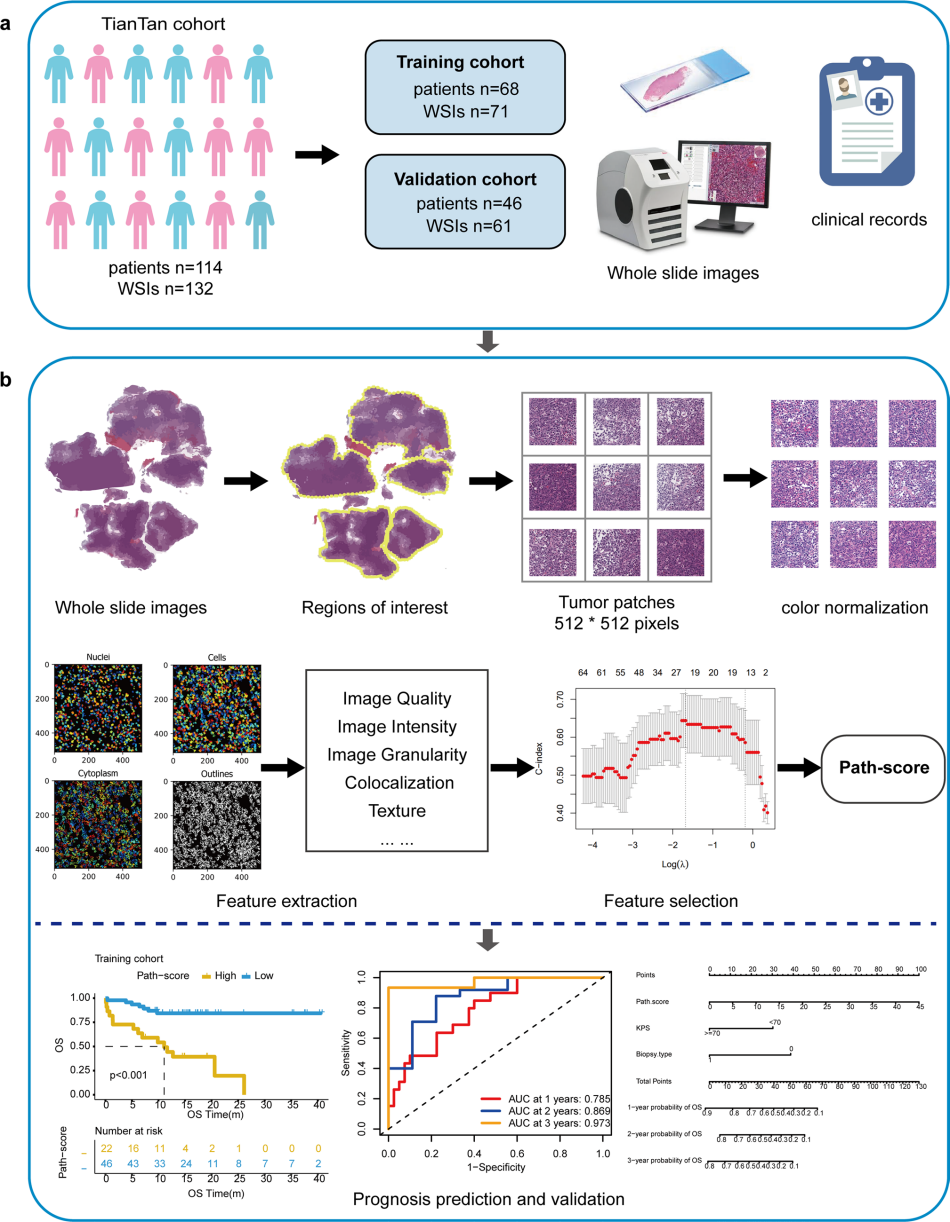
Workflow and general methodology of pathomics signature construction. **a**. Whole-slide images (WSIs) acquired from PCNSL patients are scanned. The cohort 1 contains 68 patients and corresponding 71 WSIs is used to build a prognostic model. The cohort 2 which includs 46 patients and corresponding 61 WSIs is considered as an independent validation cohort. **b.** After annotation, patch segmentation, and color normalization, multiple pathomics features are extracted from an automatic pipeline using the Cellprofiler software. The pathomics score (Path-score) is developed via the Lasso-cox regression model for each patient. Survival stratification and time-dependent receiver operating characteristic (ROC) curves are further explored. Finally, the nomogram incorporates the Path-score and clinical characteristics is constructed

The workflow of this study is illustrated in Fig. 1. For rare cancer types, especially PCNSL, data acquisition is challenging, and we cannot get data from public databases, such as the TCGA (The Cancer Genome Atlas) database. Patients with PCNSL from two cohorts at Beijing Tiantan Hospital between January 2019 and March 2023 and corresponding whole-slide images (WSIs) were retrospectively enrolled. The inclusion criteria were as follows: (1) histologically diagnosed CNS-DLBCL; (2) no other concomitant tumors; and (3) availability of complete clinicopathological and follow-up information. The exclusion criteria were as follows: (1) evidence of systemic DLBCL from computed tomography (CT) or positron emission tomography CT (PET CT) of the chest, abdomen, pelvis, and bone marrow aspiration; (2) no complete and clear WSIs; and (3) missing clinical or follow-up data. The cohort 1 contained 68 patients and corresponding 71 WSIs was used to build a prognostic model. The cohort 2 which included 46 patients and corresponding 61 WSIs was considered as an independent validation cohort (Fig. 1a).

Patient demographic information including age, sex, Eastern Cooperative Oncology Group (ECOG) PS, Karnofsky Performance Status (KPS), Hans, Biopsy type, deep lesion involvement, tumor size, number of lesions, IELSG score, MSKCC score, treatment, and response were collected. Initial treatment responses including complete response (CR), partial response (PR), stable disease (SD), and progressive disease (PD) were determined according to the International Primary CNS Lymphoma Collaborative Group criteria [26]. Patients who were in CR or PR were regarded as Responders, whereas those who were in SD or PD were categorized as Non-Responders. Patients who had progressed during the initial treatment were considered to have Primary Resistance. OS was defined as the time from diagnosis to death from any cause or last follow-up. Progression-free survival (PFS) was defined as the time from diagnosis to progression or all-cause death. This study was approved by the Institutional Review Board of Beijing Tiantan Hospital (approval number: 2020–059 YW), and written informed consent was obtained from all the patients. All the procedures complied with the standards of the Declaration of Helsinki.

### Image annotation and preprocessing of digital WSIs

All slides were formalin-fixed, paraffin-embedded, and stained with H&E. Then, the slides were scanned by a Lecia Aperio CS2 scanner into WSIs in a standard file format (the ‘svs’ format). Scanning magnification was 20X. Two highly experienced pathologists annotated the regions of interest (ROI) using ASAP (version 2.1). When the annotated results are inconsistent, a third senior pathologist will make the final judgment. The ROI patches (512 × 512 pixels) were tiled using OpenSlide and color-normalized using the Vahadane method [27] (Fig. 1b). To reduce the computational time, 50 non-overlapping representative patches that contained more tumor cells from each patient were selected by experienced pathologists for further analysis.

### Extraction of quantitative features from images

An automated feature extraction pipeline was developed using CellProfiler (version 4.2.6), an open-source image analysis software [28]. CellProfiler can quantify a variety of biological features, including basic features (e.g., cell counts and cell size) and complex morphological features (e.g., cell shape, distribution of pixel intensity in cells and nuclei, and textures of cells and nuclei). First, the images were split into hematoxylin-stained and eosin-stained greyscale images by the “UnmixColors” module. The nuclei of tumor cells were identified with the “IdentifyPrimaryObjects” module. Then the “IdentifySecondaryObjects” module identified the cell body by using the nuclei as a "seed" region, growing outwards until stopped by the image threshold or by a neighbor. Thus it identified the cytoplasm by "subtracting" the nuclei objects from the cell objects using the “IdentifyTertiaryObjects” module. The quantitative features were extracted with modules including “Measure Image Quality,” “Measure Image Intensity,” “Measure Granularity,” “Measure Colocalization,” “Measure Object Intensity,” “Measure Object Neighbors,” “Measure Object Size Shape,” and “Measure Texture” (Fig. 1b). A variety of features were measured for each identified cell or subcellular compartment, which have been proven to be valuable in characterizing microscopic cell morphology [29]. CellProfiler measures various metric features and calculates their distributions. Further description of the pipeline for feature extraction is described in the Supplementary Methods. A summary of pathological features is presented in Supplementary Table S1. The final value of each feature was averaged over 50 patches for further analyses.

### Machine-learning methods for diagnosis classification

Six common machine-learning classifiers were applied in our study: Logistic, K-Nearest Neighbor (KNN), Random Forest (RF), Support Vector Machines (SVM), eXtreme Gradient Boosting (XGBoost), and Decision Tree (DT). Models were trained and tested using R software (version 4.3.1), with “caret” package for normalization, package “mlr3’ for Logistic and KNN, package “randomForest” for RF, package “e1071” for SVM, package “xgboost” for XGBoost and package “rpart” for DT. The datasets were randomly divided into a 60% training set and a 40% test set. The Receiver Operator Characteristics (ROC) curves were plotted using “pROC” package. Area Under Curve (AUC), Accuracy, and F1 score were used to evaluate model performance.

### Feature selection and pathomics score building

A three-step feature selection procedure was applied to the training cohort to establish a pathomic signature. First, a univariate Cox regression analysis was performed to examine the prognostic value of the 802 features. Only features with *p* < 0.05 were identified as candidate prognostic features. The LASSO-Cox regression method was used to further select important features. L1 penalty tuning parameter lambda (λ) was applied to shrink the coefficients of each feature to zero. Features with non-zero coefficients were screened. In this study, tenfold cross-validation was conducted to determine the optimal λ value by measuring the concordance index (C-index) in the training cohort. Finally, we developed a multivariate Cox proportional hazards model using a backward stepwise approach. The Path-score was generated via a linear combination of selected features weighted by their respective coefficients, and the Path-score for the validation cohort was calculated using the formula obtained in the training cohort. Several packages containing “glmnet,” “survival,” and “survminer” were used in this process.

### Association of the Path-score with prognosis and clinical characteristics

The optimal cutoff value for the Path-score was determined using the maximally selected rank statistics. Patients were classified into high- and low-risk groups in the training and validation cohorts, according to the same threshold. Potential associations of the Path-score with OS and PFS were first assessed in the training cohort and then validated in the validation cohort using Kaplan–Meier survival analysis. The predictive ability of the score was assessed by the “timeROC” package. To confirm its independent prognostic value, univariate and multivariate Cox survival analyses were performed for the clinicopathological factors. The association between the Path-score and initial treatment response was assessed in the entire cohort combined with cohort 1 and cohort 2.

### Construction and assessment of the incremental value of pathomics nomogram

The nomogram incorporated the Path-score and independent clinical factors based on multivariate Cox analysis using a backward stepwise approach. To evaluate discrimination performance, the C-index and 1-, 2-, and 3-year AUROC were calculated. Calibration curves were generated to compare the predicted survival with the actual survival. Decision curve analysis (DCA) was used to assess the clinical usefulness of the nomogram by quantifying its net benefits. To compare the usefulness of the nomogram with others, the net reclassification improvement (NRI) and Integrated Discrimination Improvement (IDI) were calculated. The discrimination, calibration, and clinical usefulness assessments were validated in the validation cohort. Several packages including “rms,” “riskRegression,” “timeROC,” “dcurves,” and “survIDINRI” were used in the analysis.

### Statistic analysis

The Student’s t-test, Wilcoxon’s test, and Kruskal–Wallis test were used to compare continuous variables. The Shapiro–Wilk test was used to test the normality of data distributions. Pearson’s chi-squared test and Fisher’s exact test were used to compare categorical variables. Survival curves were generated using the Kaplan–Meier method and compared using the log-rank test. Univariate and multivariate analyses were performed using the Cox proportional hazards model. The proportional hazard assumption was tested using the Schoenfeld Individual Test. The comparisons of AUROCs and C-indexes between models were performed by using the DeLong test and z-score test, respectively. All tests were two-sided, and statistical significance was set at *p* < 0.05. All statistical analyses were performed using the R software (version 4.3.1) and Python (version 3.11.3).

## Results

### Clinicopathological characteristics in the study cohort

The clinicopathological characteristics of the combined cohort (*n* = 114), the training cohort (*n* = 68), and the validation cohort (*n* = 46) are listed in Supplementary Table S2. The two cohorts were balanced. Among the 114 patients included in this study, 57 (50.00%) were men, and the median (interquartile range (IQR)) age of all patients was 64 (54–69) years. Twelve (10.53%) patients underwent surgical resection or open biopsy, and 102 patients (89.47%) underwent stereotactic biopsy. Based on the Hans algorithm, 32 cases (28.07%) were regarded as the non-GCB subtype and 82 cases (71.93%) as the GCB subtype. Eighty-five patients (74.56%) had deep brain involvement (corpus callosum, basal ganglia, periventricular region, brainstem, and/or cerebellum). Multifocal lesions were observed in 67 (58.77%) patients. Most of these lesions were small, with 24.56% (*n* = 28) larger than 5 cm. Of all the patients, 14 (12.28%) received chemotherapy (CT) combined with radiotherapy (RT), 42 (36.84%) received BTK inhibitors therapy, and 32 (28.07%) received consolidation therapy. After the initial treatment, 61 patients (58.10%) achieved CR, 9 patients (8.57%) achieved PR, 3 patients (2.85%) experienced SD, 32 patients (30.48%) had PD, and the data for the remaining 9 patients were not available. The median OS of the combined cohort was 34.07 months (95%CI: 24.50-Not Reached (NR)) and the median PFS was 12.70 months (95%CI: 9.20–23.30) (Supplementary Fig. 1a-b).

### Image features accurately distinguish tumor tissues

After eliminating futile features, 802 quantitative features were extracted from each slide using the automated pipeline with CellProfiler (see Methods for details). To demonstrate the biological significance of these features, we utilized six machine-learning methods to explore whether these features can distinguish tumors from normal adjacent tissues. The AUC of all classifiers in the training set was higher than 0.9 (Supplementary Fig. 1c and Table S3). Our classifiers achieved an average AUC of 0.968 in the testing cohort (Classifiers: Logistic, AUC = 0.965, 95%CI: 0.917–0.996; KNN, AUC = 0.978, 95%CI: 0.964–0.999; XGBoost, AUC = 0.987, 95%CI: 0.982–1.000; SVM, AUC = 0.990, 95%CI: 0.982–1.000; Decision Tree, AUC = 0.891, 95%CI: 0.771–0.913; RF, AUC = 0.994, 95% CI: 0.982–1.000) (Supplementary Fig. 1d and Table S3). The top quantitative features selected by RF were Image Granularity, Image colocalization, Haralick features, Image Quality, and Object Intensity features (Supplementary Table S4).

### Establishment of pathomics score and its prognostic value

**Fig. 2:**
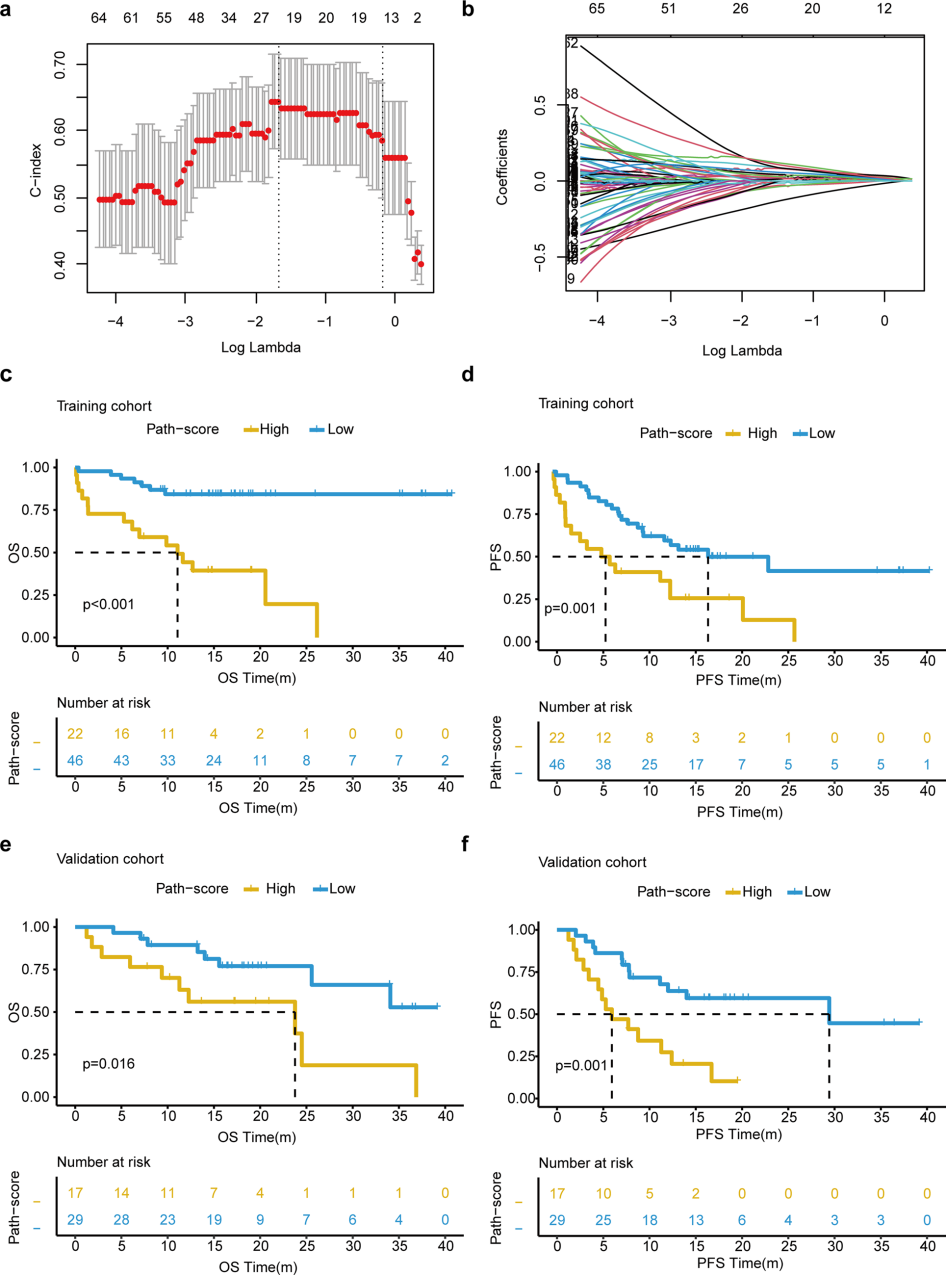
Construction and survival stratification of the pathomics score (Path-score). **a**. Pathomics features selection using the LASSO-Cox regression model via tenfold cross-validation. The x-axis is the value of log (λ) and the y-axis is the C-index. Solid vertical lines represent the partial likelihood of deviance ± SE. The dotted vertical line is shown at the optimal partial likelihood of deviance. A λ value of 0.186618, with a log (λ) value of -1.679, is chosen by tenfold cross-validation and the minimum criteria. Twenty-two features with nonzero coefficients are selected. **b.** Profiles of coefficients from the LASSO-Cox regression model of the extracted features. The figure showed the feature coefficient change with the tuning of λ value. **c.** Kaplan–Meier survival analysis for overall survival (OS) between the high and low Path-Score patients in the training cohort. *p* < 0.001 by log-rank test. **d.** Kaplan–Meier survival analysis for progression-free survival (PFS) between the high and low Path-Score patients in the training cohort. *p* = 0.001 by log-rank test. **e.** The OS difference in the validation cohort. *p* = 0.016 by log-rank test. **f.** The PFS difference in the validation cohort. *p* = 0.001 by log-rank test

In the training cohort, 91 features (*p* < 0.05) in the univariate Cox regression analysis were identified as candidate features (Supplementary Table S5). The LASSO-Cox regression model with tenfold cross-validation was then applied to further screen informative features. A λ value of 0.186618, with a log (λ) value of -1.679, is chosen by tenfold cross-validation and the minimum criteria. Thus, twenty-two features with nonzero coefficients were selected (Fig. 2a-b). Multivariate analyses using a backward stepwise approach were performed to develop the final eight-feature Path-score. The analysis met the proportional hazard assumption based on Schoenfeld Individual Test results, which showed that each covariate was not statistically significant (Global Schoenfeld Test, *p* = 0.230; Supplementary Fig. 2a). The Path-score calculation formula was: Path-score = Granularity_3_Hematoxylin*2.34096 + Mean_Cells_AreaShape_Zernike_7_1*0.01491 + Mean_Cytoplasm_AreaShape_Zernike_4_2*5.14011 + Mean_Cytoplasm_Texture_SumVariance_Hematoxylin_3_00_256*0.29709 + Mean_Nuclei_AreaShape_Zernike_1_1*4.69242 + Mean_Nuclei_Intensity_MaxIntensity_Hematoxylin*3.34853 + Mean_Nuclei_Intensity_MinIntensityEdge_Hematoxylin*0.04227 + Texture_SumVariance_Hematoxylin_3_01_256*3.38650. The Path-score of the validation cohort was acquired directly from the formula.

Patients were stratified into high and low groups, with an optimal cut-off value of 1.824 selected by maximally selected rank statistics. In the training cohort, patients with high Path-score had significantly shorter OS than low Path-score (median OS: 11.10 months, 95% CI: 6.17–NR vs. NR months, 95% CI: NR–NR, *p* < 0.001, Fig. 2c). As shown in Fig. 2d, compared with patients with low Path-score, worse PFS could be observed in patients with high Path-score (median PFS: 16.80 months, 95% CI: 9.80-NR vs. 5.72 months, 95% CI: 2.00-NR, *p* = 0.001). The same analyses were performed in the validation cohort. Among high-score patients, the median OS and PFS were 23.80 (95%CI: 11.3-NR) and 5.93 (95%CI: 4.47-NR) months, respectively. Significantly better median OS and PFS of NR (95%CI: 25.60-NR) and 29.43 (95%CI: 12.00-NR) months were found in patients with low Path-scores (Fig. 2e, log-rank *p* = 0.016; Fig. 2f, log-rank *p* = 0.001). Forest plots revealed the prognostic risk in different subgroups. Notably, the Path-score remained an effective predictor of patient survival (Supplementary Fig. 2b, HR > 1, *p* < 0.05). The distribution of the Path-score, survival status, and selected features are shown in Supplementary Fig. 3a-b, which demonstrated that a higher Path-score was associated with a higher risk of progression or death. Time-dependent ROC curves demonstrated that during the 1-, 2-, and 3-year follow-ups, the AUC values were 0.785 (95%CI: 0.668–0.902), 0.869 (95%CI: 0.730–1.000), and 0.973 (95%CI: 0.927–1.000) in the training cohort, respectively (Supplementary Fig. 3c). In the validation cohort, the AUC for 1-, 2-, and 3-year OS were 0.649 (95% CI: 0.443–0.855), 0.679 (95%CI: 0.445–0.913), and 0.733 (95%CI: 0.506–0.960), respectively (Supplementary Fig. 3d). In addition, five features revealed independent prognostic value among the final eight features (Supplementary Fig. 3e, *p* < 0.05).

### Remarkable correlation between the Path-score and treatment response

**Fig. 3:**
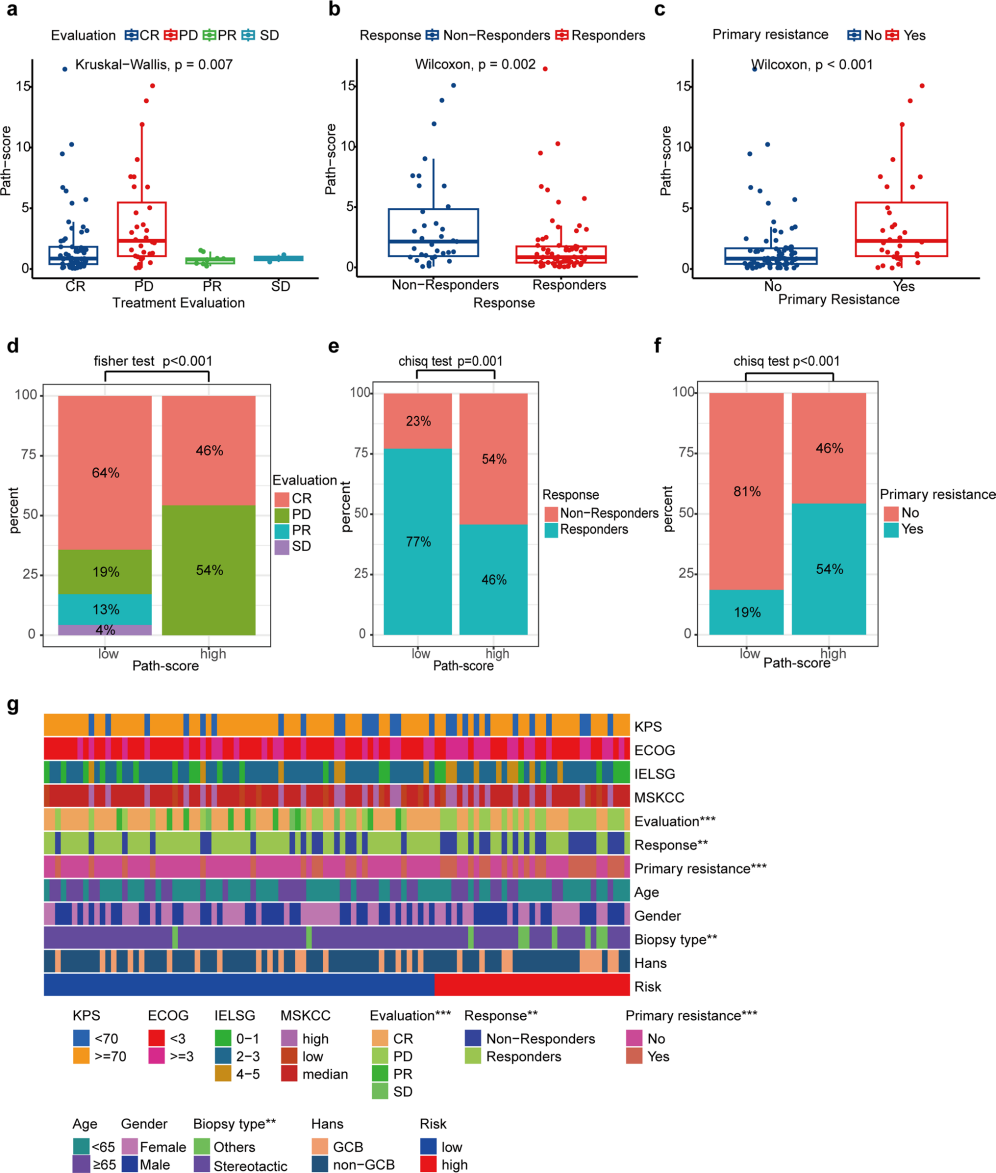
The correlation between the Path-score and clinical characteristics among the combined cohort. **a**. The distribution of Path-score based on the treatment evaluation in the combined cohort. Patients with the best response to progression disease (PD) after initial treatment show higher Path-scores than others. *p* = 0.007 by the Kruskal–Wallis test. **b.** Comparisons of the Path-score value in Responders and Non-responders. *p* = 0.002 by the Wilcoxon test. **c.** The distribution of Path-score based on primary tumor resistance. *p* < 0.001 by the Wilcoxon test. **d.** Distribution of patients in complete remission (CR)/partial remission (PR)/stable disease (SD)/PD between Path-score groups. **e.** Distribution of Non-Responders/Responders between Path-score groups. A significantly lower response rate to initial treatment is found in the high Path-score group compared to the low Path-score group. **f.** Distribution of patients who are primarily resistant to treatment or not between Path-score groups. A significantly higher rate of primary tumor resistance is found in the high Path-score group than the low Path-score group. **g.** Heatmap shows the relationship between Path-score group and each clinical characteristic. Risk represents the grouping of patients based on Path-score. ** *p* < 0.01; *** *p* < 0.001

Next, we systematically evaluated the correlation between the Path-score and other clinical characteristics of the combined cohort. As shown in Supplementary Table S6, we observed that patients with KPS < 70 (1.678 vs. 0.867, *p* = 0.028) or ECOG >  = 3 (1.646 vs. 0.883, *p* = 0.037) had a higher Path-score count than their counterparts. Therapeutic response data for 105 patients were available. Figure 3a shows the distribution of the best response according to Path-score in patients. Patients with the best response of PD after initial treatment showed higher Path-scores compared with patients with CR/PR/SD (PD: 2.313, IQR 1.052–5.467; SD: 0.859, IQR 0.719–1.014; PR: 0.775, 0.462–0.875; CR: 0.858, IQR 0.396–1.824; Kruskal–Wallis *p* = 0.007)**.** In particular**,** when considered as a continuous variable, patients who responded to initial treatment had lower Path-score than Non-Responders (median Path-score in Responders: 0.849, IQR: 0.403–1.743 vs. median Path-score in Non-Responders: 2.150, IQR: 0.942–4.833; *p* = 0.002) (Fig. 3b). Path-score was higher in patients with primary tumor resistance (median Path-score: 2.313, IQR: 1.052–5.467 vs. median Path-score: 0.852, IQR: 0.414–1.704 in patients not experiencing primary resistance; *p* < 0.001) (Fig. 3c).

Moreover, we also analyzed the differences in clinical characteristics between Responders and Non-Responders. Non-responders to treatment include more people in high Path-score groups (19/35, 54.29% vs. 16/70, 22.86%; *p* = 0.003, Supplementary Table S7). Other clinical factors were not found to be associated with patient response to treatment. Up to 54% of patients in the high Path-score group experienced PD, which was significantly higher than that in the low Path-score group (Fig. 3d, *p* < 0.001). Accordingly, a significantly lower response rate to initial treatment was found in patients in the high Path-score group (19/35, 54.29%) as compared to patients in the low Path-score group (16/70, 22.86%; *p* < 0.001, Fig. 3e). High Path-score was associated with primary resistance to therapy (high Path-score group: 19/35 patients, 54.29%; low Path-score group: 13/70 patients, 18.57%; *p* < 0.001, Fig. 3f). Heatmap summarized the relationship between Path-score group and each clinical characteristic (Fig. 3g). Based on the above results, we further explored the predictive performance of Path-score on disease treatment response, primary resistance, and disease recurrence. As shown in Supplementary Fig. 4a, the AUC values of the Path-score predicting treatment response were 0.684 (95%CI: 0.574–0.794). Our pathomics signature could accurately distinguish primary resistance from patients, with AUC value of 0.706 (95%CI: 0.593–0.819, Supplementary Fig. 4b). Also, it was possible to predict recurrence with AUC value of 0.618 (95%CI: 0.505–0.732, Supplementary Fig. 4c). Patients with disease recurrence showed higher Path-score (1.337, IQR 0.699–4.063 vs. 0.884, IQR 0.393–1.823, *p* = 0.042). These results confirmed that the Path-score was significantly correlated with the patient’s response to treatment.

### Development and validation of a nomogram

In the univariate Cox regression analysis, the Path-score, Biopsy type, KPS, ECOG PS, and IELSG were significantly associated with OS in the training cohort. (Supplementary Table S8, *p* < 0.05). Multivariate Cox regression analysis was performed adjusting for clinicopathological variables. High Path-score were independently associated with OS (HR 7.423, 95%CI: 2.738–20.119, *p* < 0.001) and PFS (HR 2.143, 95%CI: 1.116–4.113, *p* = 0.022) in the training cohort. It remained a powerful and independent prognostic factor for predicting OS and PFS in the validation cohort according to the multivariate Cox regression analysis (OS: HR 4.204, 95%CI: 1.299–13.601, *p* = 0.017; PFS: HR 3.243, 95%CI: 1.440–7.301, *p* = 0.005; Supplementary Table S9).

**Fig. 4:**
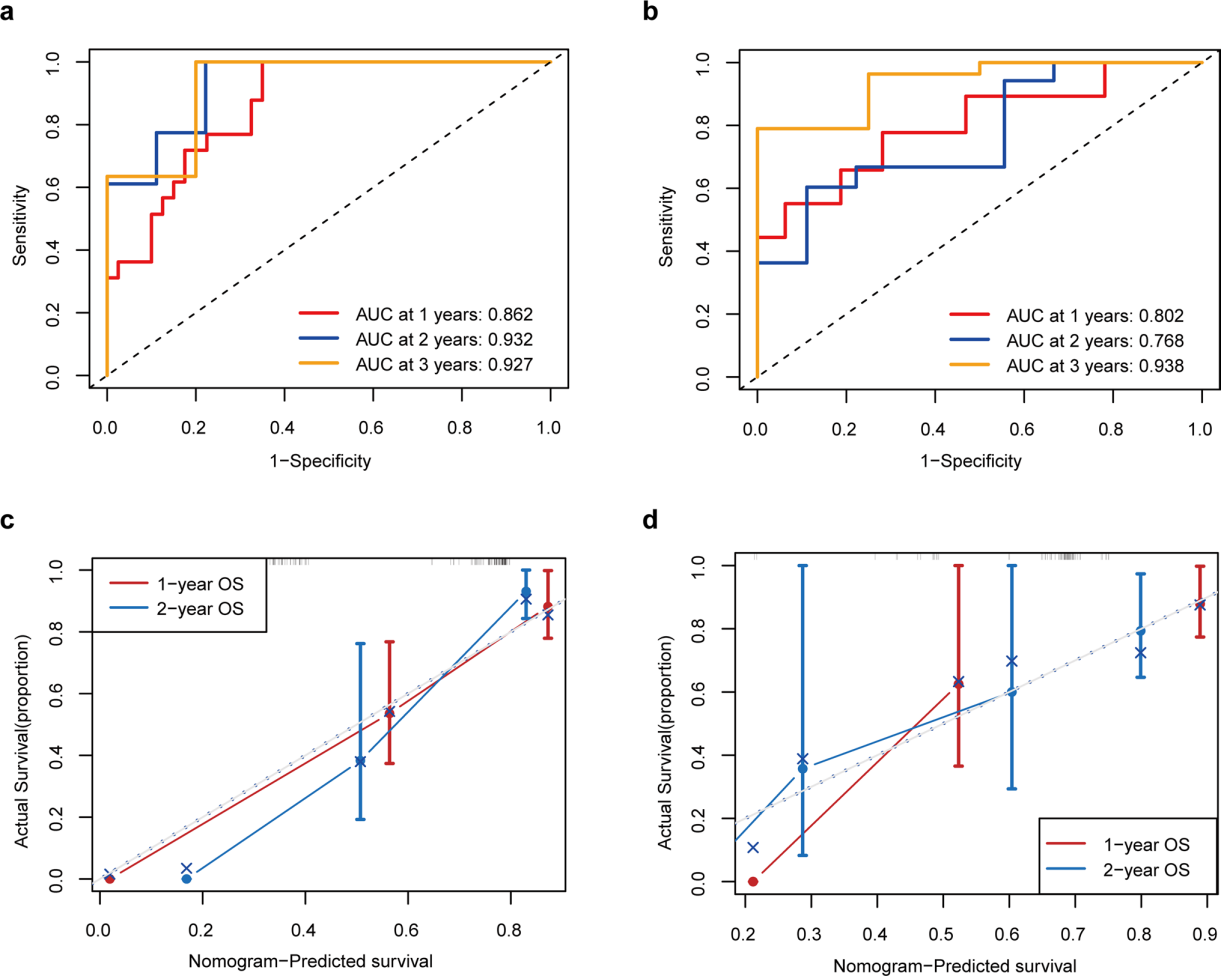
Performance of the pathomics nomogram for the prediction of overall survival. **a**. The time-independent ROC curves of nomogram in the training cohort. **b.** The time-independent ROC curves of nomogram in the validation cohort. **c.** The calibration curves of the nomogram between predicted and actual 1- and 2-year OS in the training cohort. **d.** The calibration curves of the nomogram between predicted and actual 1- and 2-year OS in the validation cohort. Solid vertical lines and error bars represent the mean agreement between nomogram-predicted survival and actual probability of survival and the corresponding 95% confidence interval, respectively

Backward stepwise multivariate Cox regression analysis demonstrated that the Path-score, KPS, and Biopsy type were independently associated with OS (Supplementary Fig. 5a, *p* < 0.05). To improve the accuracy of OS prediction for PCNSL patients, we developed an integrated nomogram by combining Path-score and predictable clinical factors, including Biopsy type and KPS. The integrated nomograms for 1-year, 2-year, and 3-year OS prediction are shown in Supplementary Fig. 5b. The C-index of the nomogram in the training and validation cohorts were 0.849 (95%CI: 0.790–0.908) and 0.747 (95%CI: 0.608–0.886), respectively. In addition, the time-dependent ROC curve of the nomogram at 1-, 2-, and 3-year depicted AUC of 0.862 (95%CI: 0.772–0.953), 0.932 (95% CI: 0.835–1.000), and 0.927 (95%CI: 0.787–1.000) for OS, respectively (Fig. 4a). In the validation cohort, it showed an improved AUC for 1-, 2-, and 3-year OS were 0.802 (95%CI: 0.624–0.980), 0.768 (95%CI: 0.576–0.960), and 0.938 (95%CI: 0.837–1.000), respectively (Fig. 4b). Furthermore, the calibration curves showed a favorable agreement between the nomogram-predicted survival and actual survival in both the training and validation cohorts (Fig. 4c-d).

### Incremental value of the nomogram in survival prediction

**Fig. 5:**
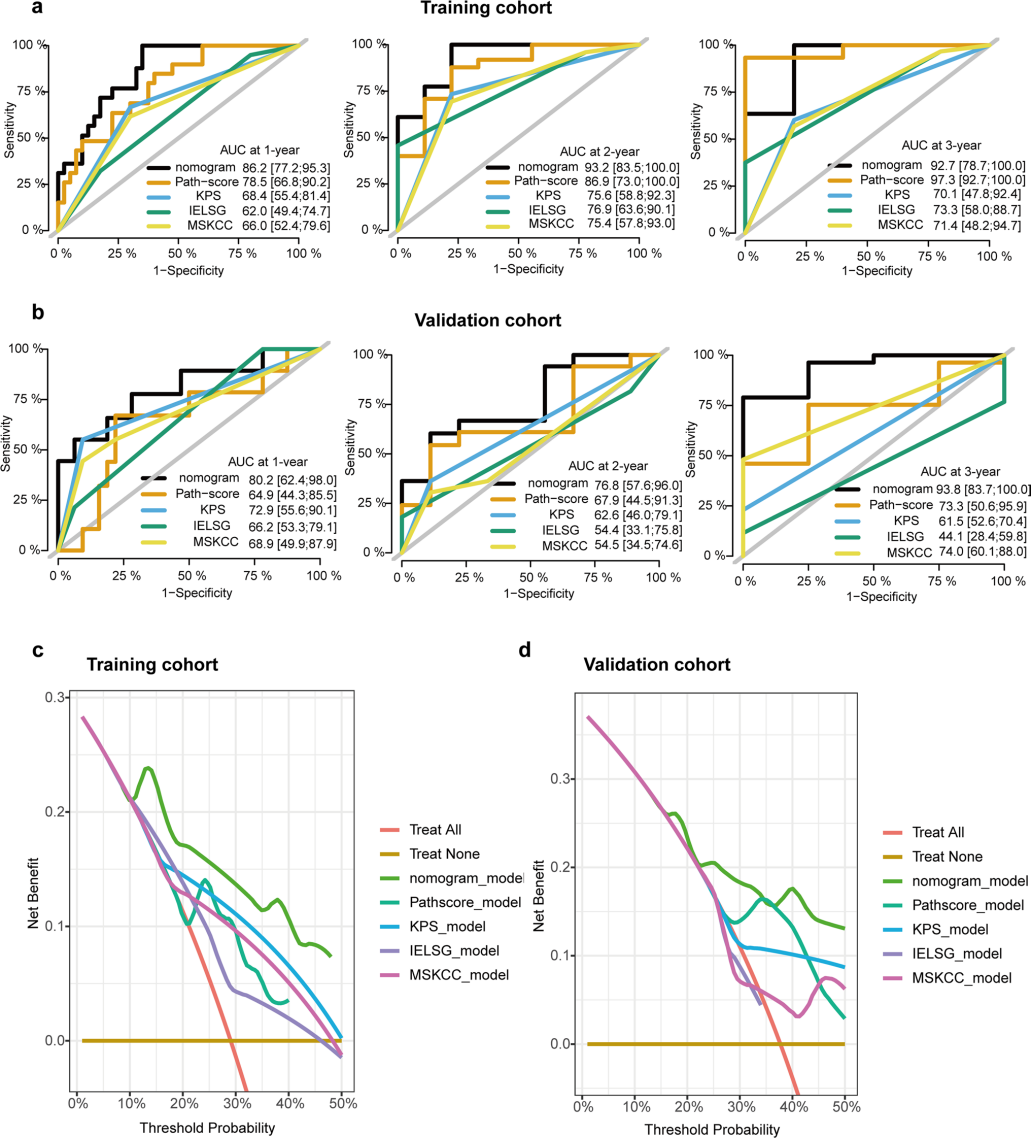
The incremental value of the pathomics nomogram. **a**. AUROC comparisons between the pathomics nomogram and other models at 1-year, 2-year, and 3-year overall survival (OS) in the training cohort. **b.** AUROC comparisons between the pathomics nomogram and other models at 1-year, 2-year, and 3-year OS in the validation cohort. The comparisons of AUCs between two models are performed using a two-sided Delong test. **c.** Decision curve analysis of OS for different models in the training cohort. **d.** Decision curve analysis of OS for different models in the validation cohort. The y-axis measures the net benefit. The net benefit was calculated by summing the benefits (true positive results) and subtracting the harms (false positive results). The pathomics nomogram had the highest net benefit compared to both the other models

The C-index of the Path-score for the prediction of OS in the training cohort and validation cohort was 0.745 (95%CI 0.639–0.851) and 0.623 (95%CI: 0.472–0.774), respectively. Compared with the Path-score alone, the pathomics nomogram displayed a significantly improved C-index of 0.849 (95%CI: 0.790–0.908, *p* = 0.001) in the training cohort and 0.747 (95%CI: 0.608–0.886, *p* = 0.009) in the validation cohort, respectively (Supplementary Table S10). Similarly, the AUCs of the Path-score for 1-, and 2-year OS were 0.785 (95%CI: 0.668–0.902) and 0.869 (95%CI: 0.730–1.000), respectively. Compared with it, the nomogram exhibited a significantly higher AUC of 0.862 (95%CI: 0.772–0.953; *p* = 0.017) and 0.932 (95%CI: 0.835–1.000; *p* = 0.040), respectively (Fig. 5a, Supplementary Table S11). Similar results were validated in the validation cohort. The nomogram showed better predictive capability for 1-, 2-, and 3-year OS than Path-score (1-year OS: nomogram vs. Path-score, 0.802 (95%CI: 0.624–0.980) vs. 0.649 (95%CI: 0.443–0.855), *p* = 0.068; 2-year OS: nomogram vs. Path-score, 0.768 (95%CI: 0.576–0.960) vs. 0.679 (95%CI: 0.445–0.913), *p* = 0.425; 3-year OS: nomogram vs, Path-score, 0.938 (95%CI: 0.837–1.000) vs. 0.733 (95%CI: 0.506–0.959); *p* = 0.033) (Fig. 5b**, **Supplementary Table S11). Meanwhile, the predicted AUC of the IELSG model for 1-year (AUC 0.620, 95%CI: 0.494–0.747, *p* = 0.046), 2-year (AUC 0.769, 95%CI: 0.636–0.901, *p* < 0.001), and 3-year OS (AUC 0.733, 95%CI: 0.580–0.887, *p* = 0.001) were significantly worse than nomogram in the training cohort (Fig. 5a**, **Supplementary Table S12). Consistently, the predictive performance of the MSKCC model was also worse than the nomogram (Fig. 5a**, **Supplementary Table S13). The abovementioned results were well validated in the validation cohort (Supplementary Tables S12-13). DCA was performed to evaluate the clinical decision utility of the nomogram. The combined nomogram also showed a higher overall net benefit than the Path-score, KPS, IELSG, and MSKCC in the training and validation cohorts (Fig. 5c-d). Furthermore, the pathomics nomogram exhibited an NRI of 0.469 (95% CI: 0.157–0.643; *p* = 0.004) and an IDI of 0.152 (95%CI: 0.059–0.308; *p* < 0.001) compared to the Path-score in the training cohort (Supplementary Table S14). An NRI of 0.457 (95% CI: 0.082–0.767; *p* = 0.016) and IDI of 0.229 (95% CI: 0.047–0.501; *p* = 0.004) for OS were also observed in the validation cohort (Supplementary Table S14). Consequently, the combined nomogram showed incremental performance and improved classification accuracy for survival outcomes compared with the other models.

## Discussion

To our knowledge, this is the first study to investigate the utility of a predictive model in PCNSL patients by the quantitative histopathological features extracted from whole-slide pathology images. In the current study, we constructed an eight-feature-based pathomics signature to predict the outcomes of patients with PCNSL, which successfully stratified patients into high- and low-score groups with significant differences in OS and PFS, and was confirmed to be an independent prognostic factor. Moreover, by incorporating the pathomics signature with clinical characteristics, we developed and validated a pathomics nomogram that exhibited improved discrimination and calibration.

Although the outcomes of patients with PCNSL have significantly improved with advances in initial treatment, many patients still die because of relapse or chemotherapy-resistant disease [30]. The most commonly used IELSG and MSKCC prognostic models were developed over a decade ago [13, 14], and most recent studies have failed to validate them [31–33]. Several prognostic models regarding clinical and laboratory parameters have been proposed for PCNSL in the past few years, but a consensus on the optimal model for patients is lacking. Thus, it is crucial to develop an innovative and reliable predictive model that will provide an accurate prognosis for patients and allow appropriate therapeutic decision-making. With the rapid development of innovative technologies and requirements of precision medicine, medical imaging has transformed from a simple diagnostic tool to an enormous source of clinical data. Two new representative research fields including “radiomics” and “pathomics” have attracted increasing attention. The vast amount of information contained in WSIs is available to assist oncologists in detecting hidden information based on advances in digital pathology [34]. Pathomics is a novel method that has proven to be effective in tumor diagnosis, classification, and survival prediction in several highly prevalent common cancer types, such as malignant lymphoma [35], glioma [18], hepatocellular carcinoma [36], and colorectal cancer [37]. However, for rare cancer types, especially PCNSL, the study is still very limited. Currently, almost all published studies related to PCNSL have focused on the differentiation of PCNSL from glioblastoma (GBM) [38, 39]. Chen et al. evaluated the prognostic value of texture features on contrast-enhanced magnetic resonance imaging (MRI) in 52 patients with PCNSL. To date, the literature regarding the prognostic prediction of digital pathology analysis in PCNSL is yet to be reported. Herein, as the first attempt, to the best of our knowledge, we discovered that the pathomics signature extracted from pathology images could also contribute to the prediction of prognosis in PCNSL. The Path-score, containing eight features selected by the Lasso-Cox regression model, was observed to be significantly associated with OS and PFS. As H&E-stained slides are routinely used in the clinic, the present pathomics signature, which was derived from pathology slides, might be a noninvasive, convenient, low-cost, and reproducible approach to characterize tumor phenotypes.

Despite therapeutic progress in the treatment of PCNSL, approximately 15–25% of patients do not respond to HD-MTX-based chemotherapy and up to half of patients relapse after the initial response [40]. Patients with primary refractory disease or relapse exhibit poor prognosis, with a median survival of 2 months without additional treatment [6, 41]. Relapse-acquired drug resistance after HD-MTX treatment remains a serious challenge. A recent metabolomic profiling demonstrated that glycolysis was excessive via PI3K/AKT/mTOR and RAS/MAPK Signaling in methotrexate-resistant PCNSL-derived cells, which is valuable to understanding targeted therapies with selective anticancer drugs in recurrent CNS lymphoma [42]. Accurate biomarkers that can identify patients who are likely to benefit from the initial treatment will improve their prognostic ability and personalized therapy. Lin et al. reported that NK cells in the peripheral blood have an impact on the outcome and chemotherapy benefits of PCNSL [43]. In the present study, we showed that patients who achieved CR/PR had a lower Path-score than those who achieved SD/PD. The high Path-score group included more patients who progressed during the initial treatment, while the low Path-score group comprised more patients who responded to treatment. Thus, patients with higher Path-score had a higher likelihood of progression. Additionally, our pathomics signature demonstrated good predictive performance for treatment response, primary resistance, and disease recurrence. Our results indicated that the pathomics features reflected intratumor heterogeneity and might be a potential indicator of treatment response in patients with PCNSL.

In the present study, we demonstrated that the pathomics signature successfully identified high-risk patients with poor survival outcomes. It remained an effective predictive value after the stratified analysis of clinical characteristics, as depicted in the forest plot (Supplementary Fig. 2b, HR > 1, *p* < 0.05). Supplementary Fig. 6a-b showed some examples of histopathology images from two PCNSL patients with the same pathology subtype and similar clinical characteristics (Patient A: a 39-year-old female, with non-GCB subtype, single lesion, no deep involvement, KPS ≥ 70, ECOG < 3, IELSG 0–1, treated with HD-MTX based chemotherapy; Patient B: a 51-year-old male, with the same baseline characteristics), but with different survival outcomes. These quantitative image features are often difficult to detect through manual inspection, but computer methods can identify these features efficiently and effectively. Pathomics is a novel method, that is available to explore tumor heterogeneity since varying degrees of disease progression, clinical outcomes, and treatment response correspond to histologic features in different tumor cells [44]. Digital pathology can empower pathologists with the ability to quantitatively assess diagnostic features of cancer by providing quantitative data about different types of cells and tissue structures and calculated features of nuclei like size, area, color, chromatin density, and mitotic activity [44]. The quantitative features covered the size, shapes, pixel intensity distributions, textures of the objects, as well as the relation between neighboring objects. These features have been proved to be valuable in characterizing the microscopic cell morphology [45]. We also investigated the top features associated with prognosis in PCNSL. The primary prognostic features included Zernike shape features of the cell, nuclei, cytoplasm, texture features, and nuclei intensity. Zernike shape features were extracted by identifying the circle of the smallest diameter covering the tumor nuclei of each cell, setting all pixels within the nuclei to one and background to zero. The resulting binary image is then decomposed into Zernike polynomials, where the coefficients are used as features. Texture features quantify the correlation between nearby pixels within a region of interest. This showed that both local anatomical features (shape of cell, nuclei, and cytoplasm) and global patterns (texture of the cytoplasm) were associated with survival outcomes. Recently, comprehensive analysis of histopathological images and genomic data has provided a feasible approach to explore the potential mechanisms of pathomics signature with prognosis and therapeutic response [46, 47]. We expect that genomics, radiomics, and pathomics can be utilized together to improve the prediction of patient outcomes and therapeutic response of PCNSL in the future, thereby accelerating the development of personalized medicine.

To provide a more individualized prognostic prediction tool, a nomogram that combines pathomics and clinical features has been developed to better predict the overall survival of individual patients. The nomogram exhibited a higher predictive ability than the Path-score alone (C-index, 0.749 vs. 0.849). Higher time-AUCs, NRIs (*p* < 0.05), and IDIs (*p* < 0.05) were also observed in the novel combined nomogram. The decision curve analysis confirmed that the nomogram was superior to the Path-score, KPS, IELSG, and MSKCC models, which indicated that the combined nomogram showed incremental value for individualized prediction.

Deep learning has been proven to be a novel approach for tumor diagnosis, grading, and molecular prediction within histopathological images, which can adaptively extract image features based on learning objectives [48, 49]. Despite remarkable developments in other applications, deep learning has not yet been widely used to solve time-to-event prediction problems. Learning survival directly from histology is considerably more complex. In addition, the inherent complexity of deep neural networks often regards them as "black boxes," raising significant concerns regarding the interpretability and reproducibility of their results. In this study, we established an automated pipeline to identify tumor cells and extract a variety of features directly from images. Machine-learning models with selected features successfully distinguished tumors from adjacent normal tissues, showing that our image features can capture hidden important image labels. Moreover, CellProfiler is a versatile, open-source software designed for high-throughput image analysis and has been used in digital pathology analysis with favorable performance [28, 29]. Accordingly, CellProfiler stands as a robust and accessible solution that allows researchers to extract quantitative pathological features.

Our study has some limitations. First, the retrospective nature of the data collection may have influenced its reproducibility and generalization. This retrospective study might be subject to inherent biases and unknown confounders, although we have verified our major results in a validation cohort. Second, the sample size was relatively small, mainly because of the low incidence of PCNSL [1]. For rare cancer types, especially PCNSL, data acquisition is challenging, and we cannot get data from public databases. We have tried our best to collect all the slides we can get. We are currently trying to continue collecting pathological slides from PCNSL patients, and hope to establish a larger sample cohort in the future to further validate our findings. Third, further investigation in prospective randomized trials incorporating different populations is necessary to explore the clinical utility of the pathomics signature for individualized decision-making. Despite the requirement for a large-sample independent prospective multicenter validation cohort, the decision curve analysis in this study, which evaluates the clinical utility without additional validation data, indicates that the pathomics nomogram has considerable potential in clinical applications for patient prognosis prediction.

## Conclusion

In summary, as a non-invasive, accessible, and convenient approach, the pathomics signature can successfully predict the survival outcomes of patients with PCNSL. By integrating the pathomics signature with the clinical characteristics, we developed and validated a nomogram that adds incremental prognostic value compared to existing prognostic models. Furthermore, the pathomics signature may be a potential tool for predicting patient benefits from initial treatment.

### Supplementary Information

Below is the link to the electronic supplementary material.

Supplementary file1 (DOCX 22 KB)

Supplementary file2 (DOCX 21 KB)

Supplementary file3 (DOCX 41987 KB)

Supplementary file4 (DOCX 84 KB)

## Data Availability

No datasets were generated or analysed during the current study.
