# Supplementary material for: Machine learning-based pathomics signature of histology slides as a novel prognostic indicator in primary central nervous system lymphoma
Source: J Neurooncol. 2024 Apr 1;168(2):283–98. doi: 10.1007/s11060-024-04665-8 (PMC11147825; doi:10.1007/s11060-024-04665-8)
Supplement: Supplementary file 1 — Supplementary file1 (DOCX 22 KB) [file 11060_2024_4665_MOESM1_ESM.docx]

**Article title:** Machine learning-based pathomics signature of histology slides as a novel prognostic indicator in primary central nervous system lymphoma

**Journal name:** Journal of Neuro-Oncology

**Author names:** Ling Duan^1^, Yongqi He^1^, Wenhui Guo^1^, Yanru Du^2^, Shuo Yin^1^, Shoubo Yang^1^, Gehong Dong^2*^, Wenbin Li^1*^, Feng Chen^1*^

**Affiliations:** 1. Department of Neuro-Oncology, Cancer Center, Beijing Tiantan Hospital, Capital Medical University, Beijing, 100070, China; 2. Department of Pathology, Beijing Tiantan Hospital, Capital Medical University, Beijing, 100070, China

**Corresponding author:** Gehong Dong, E-mail: 13520157603@126.com; Wenbin Li, Email: liwenbin@ccmu.edu.cn; Feng Chen, Email: chenfeng406@sina.com.

**Supplementary Methods**

**Extraction of quantitative features from images**

An automated feature extraction pipeline was generated using CellProfiler (version 4.2.6), an open-source image analysis software developed by the Broad Institute (Cambridge, MA)^[1]^. The detailed step-by-step pipeline is provided as follows, and more details can be found at <https://cellprofiler.org/>.

First, the selected H&E-stained images were imported into the software program and then split into hematoxylin-stained and eosin-stained grayscale images using the ‘‘UnmixColors’’ module^[2]^. It identified the tissue foreground from the unstained background by a threshold calculated by the Otsu algorithm. Secondly, the “IdentifyPrimaryObjects” module with adaptive Otsu thresholds was utilized to identify the nucleus of the tumor cells. The “IdentifySecondaryObjects” module identified cell bodies by using the nuclei as a "seed" region, growing outwards until stopped by the image threshold or by a neighbor. Then cytoplasm was defined as the regions in the cell outlines but outside of nuclei outlines using the “IdentifyTertiaryObjects” module. The above steps were called “Image Preprocessing” and “Object Preprocessing”.

Next, we used the measurement module to extract features from the entire image (hematoxylin-stained/eosin-stained grayscale images) and the object (nuclei/cell/cytoplasm) respectively. The quality features were obtained based on the ‘‘MeasureImageQuality’’ module, indicating possible image aberrations^[3]^. There were two types of quality features (blur features and threshold features) in this study. And the intensity features were generated using the “MeasureImageIntensity”. The granularity features were extracted using the ‘‘MeasureGranularity’’ module. The image granularity measurement tries to fit a series of structure elements of increasing size into the texture of the image and outputs a spectrum of measures based on how well they fit. Then the ‘‘MeasureColocalization’’ module were applied to extract features that measure the colocalization and correlation between intensities in different images on a pixel-by-pixel basis across an entire image^[4]^.

After the nuclei and cytoplasm of each cell were identified, we used the “Measure Object Intensity” module to extract the intensity features of each cell/nuclei/cytoplasm. The “Measure Object Neighbours” and “Measure Object Size Shape” modules were applied to measure neighbor relations and shape features. Subsequently, the ‘‘MeasureTexture’’ module was used to extract the texture features, which measure the degree and nature of textures within images to quantify their roughness and smoothness. The texture features were derived from the co-occurrence matrix, containing information about how image intensities in pixels with a certain position about each other occur together. The quantitative features covered the size, shapes, pixel intensity distributions, textures of the objects, as well as the relation between neighboring objects. These features were shown to be valuable in characterizing the microscopic cell morphology^[5]^. Further details about all features are illustrated in **Supplementary Table S1**.

**Supplementary References**

1. Stirling DR, Swain-Bowden MJ, Lucas AM, Carpenter AE, Cimini BA, Goodman A (2021) CellProfiler 4: improvements in speed, utility and usability. BMC Bioinformatics 22: 433 doi:10.1186/s12859-021-04344-9

2. Ruifrok AC, Johnston DA (2001) Quantification of histochemical staining by color deconvolution. Anal Quant Cytol Histol 23: 291-299

3. Bray M-A, Fraser AN, Hasaka TP, Carpenter AE (2012) Workflow and metrics for image quality control in large-scale high-content screens. J Biomol Screen 17: 266-274 doi:10.1177/1087057111420292

4. Aaron JS, Taylor AB, Chew T-L (2018) Image co-localization - co-occurrence versus correlation. J Cell Sci 131 doi:10.1242/jcs.211847

5. Kamentsky L, Jones TR, Fraser A, Bray M-A, Logan DJ, Madden KL, Ljosa V, Rueden C, Eliceiri KW, Carpenter AE (2011) Improved structure, function and compatibility for CellProfiler: modular high-throughput image analysis software. Bioinformatics 27: 1179-1180 doi:10.1093/bioinformatics/btr095
