# Supplementary material for: Machine learning-based pathomics signature of histology slides as a novel prognostic indicator in primary central nervous system lymphoma
Source: J Neurooncol. 2024 Apr 1;168(2):283–98. doi: 10.1007/s11060-024-04665-8 (PMC11147825; doi:10.1007/s11060-024-04665-8)
Supplement: Supplementary file 2 — Supplementary file2 (DOCX 21 KB) [file 11060_2024_4665_MOESM2_ESM.docx]

**Article title:** Machine learning-based pathomics signature of histology slides as a novel prognostic indicator in primary central nervous system lymphoma

**Journal name:** Journal of Neuro-Oncology

**Author names:** Ling Duan^1^, Yongqi He^1^, Wenhui Guo^1^, Yanru Du^2^, Shuo Yin^1^, Shoubo Yang^1^, Gehong Dong^2*^, Wenbin Li^1*^, Feng Chen^1*^

**Affiliations:** 1. Department of Neuro-Oncology, Cancer Center, Beijing Tiantan Hospital, Capital Medical University, Beijing, 100070, China; 2. Department of Pathology, Beijing Tiantan Hospital, Capital Medical University, Beijing, 100070, China

**Corresponding author:** Gehong Dong, E-mail: 13520157603@126.com; Wenbin Li, Email: liwenbin@ccmu.edu.cn; Feng Chen, Email: chenfeng406@sina.com.

**Supplementary Codes**

##Codes for running lasso-cox regression Fig.2

library(glmnet)

lasso <- read.table("training features.csv", sep = ",", head = T,row.names=1)

y <- cbind(time=lasso$OS.time,status=lasso$OS)

x <- as.matrix(lasso[,3:93])

fit <- glmnet(x,y,family = "cox")

plot(fit,xvar = "lambda",label = T)

set.seed(1111)

cvfit <- cv.glmnet(x,y,family="cox",type.measure="C",nfolds=10)

cvfit

plot(cvfit)

coefficients<-coef(cvfit,s=cvfit$lambda.min)

coefficients<-coefficients[which(coefficients!=0),]

coefficients

##Codes for generating survival curves Fig.2

library(survminer)

library(survival)

surv<-read.csv(file="surv.csv",stringsAsFactors = FALSE,row.names = 1)

surv$PFS.time=surv$PFS.time/30

surv$OS.time=surv$OS.time/30

res.cut<-surv_cutpoint(surv,time="OS.time",event="OS",variables="Pathscore")

summary(res.cut)

res.cat<-surv_categorize(res.cut)

head(res.cat)

plot(res.cut, palette = "npg")

fit<-survfit(Surv(OS.time,OS)~Pathscore,data=res.cat)

diff=survdiff(Surv(OS.time, OS) ~Pathscore,data =res.cat)

pValue=1-pchisq(diff$chisq,df=1)

if(pValue<0.001){

pValue="p<0.001"

}else{

pValue=paste0("p=",sprintf("%.03f",pValue))

}

p<-ggsurvplot(fit,

xlab="OS Time(m)",

ylab="OS",

legend.title="Path-score",

legend.labs=c("High","Low"),

font.x=c(14,"bold.italic","black"),

font.y=c(14,"bold.italic","black"),

pval=pValue,

conf.int = FALSE,

risk.table = TRUE,

risk.table.y.text=FALSE,

tables.height = 0.3,

break.time.by = 5,

risk.table.col="strata",

linetype = 1,

surv.median.line = "hv",

palette = c("#E7B800", "#2E9FDF"))#,"#DC0000B2", "#3C5488B2"))

p

##Codes for boxplot and bar chart Fig.3

library(limma)

library(ggpubr)

ggboxplot(cli, x = "Evaluation", y = "Pathscore",

color = "Evaluation", add="jitter", palette = "jama")+

stat_compare_means(method = "kruskal.test")

ggboxplot(cli, x = "Response", y = "Pathscore",color = "Response",add="jitter",

palette = "nature",labels = c("0" = "Non-Responders","1" = "Responders"))+

stat_compare_means()

library(ggmosaic)

library(tidyverse)

library(scales)

percent.table <- cli %>%

group_by(Pathscore, Response) %>%

summarise(count = n()) %>%

mutate(percent = count/sum(count)*100)

percent.table<-percent.table[1:4,]

percent.table %>%

ggplot(aes(Pathscore, percent,fill = Response))+

geom_col()+

geom_text(aes(label = paste0(round(percent),"%")),

position = position_stack(vjust = 0.5),

size = 5)+

theme_bw()+

theme(text = element_text(size = 12))

mytable<-table(cli$Pathscore,cli$Response)

chisq.test(mytable,correct = T)

fisher.test(mytable)

##Codes for development and validation of nomogram

training_data <- read.table("training data.csv", sep = ",", head = T,row.names=1)

validation_data <- read.table("validation data.csv", sep = ",", head = T,row.names=1)

library(rms)

library(survival)

dd_training <- datadist(training_data)

options(datadist="dd_training")

#Codes for constructing and plotting nomogram

f_OS <- cph(Surv(OS.time,OS) ~ Pathscore+KPS+Biopsytype, data = training_data, x = T, y = T, surv = T)

surv_OS <- Survival(f_OS)

nom_OS <- nomogram(f_OS, fun = list(function(x) surv_OS(12, x),

function(x) surv_OS(24, x),

function(x) surv_OS(36, x)),

fun.at = c(seq(.1,.9,by = .1),.95),

funlabel = c("1-year probability of OS","2-year probability of OS","3-year probability of OS"),lp = 0)

plot(nom_OS)

#Codes for calculating C-index in training cohort

rcorrcens(Surv(OS.time,OS) ~ predict(f_OS), data = training_data)

#Codes for calculating C-index in validation cohort

f_OS_val <- cph(Surv(OS.time,OS)~predict(f_OS, newdata = validation_data), x = T, y = T, surv = T, data = validation_data)

rcorrcens(Surv(OS.time,OS) ~ predict(f_OS, newdata = validation_data), data = validation_data)

#Codes for calibration curve Fig.4

cal<-calibrate(f,u=12,cmethod='KM',method="boot",m=15,b=100)

plot(cal)

abline(0,1,lty=3,lwd=2,col=c(rgb(0,0,255,maxColorValue= 255)))

cal<-calibrate(f,u=24,cmethod='KM',method="boot",m=5,b=50)

plot(cal)

abline(0,1,lty=3,lwd=2,col=c(rgb(0,0,255,maxColorValue= 255)))

cal<-calibrate(f,u=36,cmethod='KM',method="boot",m=2,b=50)

plot(cal)

abline(0,1,lty=3,lwd=2,col=c(rgb(0,0,255,maxColorValue= 255)))

#Codes for time-independent ROC curve and AUROC comparison Fig.4-5

library(riskRegression)

library(survival)

library(rms)

Srv_OS <- Surv(cox$OS.time, cox$OS)

coxmod_nomogram <- coxph(Srv_OS ~ Pathscore+KPS+Biopsytype, data=cox,x=TRUE)

coxmod_Pathscore <- coxph(Srv_OS ~ Pathscore, data=cox,x=TRUE)

coxmod_KPS <- coxph(Srv_OS ~ KPS, data=cox,x=TRUE)

coxmod_IELSG <- coxph(Srv_OS ~ IELSG, data=cox,x=TRUE)

coxmod_MSKCC <- coxph(Srv_OS ~ MSKCC, data=pivcox,x=TRUE)

ROC_training_OS<-Score(list("nomogram"=coxmod_nomogram,"Path-score"=coxmod_Pathscore,"KPS"=coxmod_KPS,"IELSG"=coxmod_IELSG,"MSKCC"=coxmod_MSKCC),formula=Hist(OS.time,OS)~1,data=cox,times=36,plots="ROC",metrics="auc")

ROC_training_OS

plotROC(ROC_training_OS)

#Codes for C-index comparison

library(compareC)

f_OS_nomogram <- cph(Surv(OS.time,OS) ~ Pathscore+KPS+Biopsytype, data = training_data, x = T, y = T, surv = T, time.inc = 12)

rcorrcens(Surv(OS.time,OS) ~ predict(f_OS_nomogram), data = training_data)

f_OS_Pathscore <- cph(Surv(OS.time,OS) ~ Pathscore, data = training_data, x = T, y = T, surv = T, time.inc = 12)

rcorrcens(Surv(OS.time,OS) ~ predict(f_OS_Pathscore), data = training_data)

compareC(timeX = training_data$OS.time, statusX = training_data$OS,

scoreY = predict(f_OS_nomogram), scoreZ = predict(f_OS_Pathscore))

#Codes for decision curve analysis Fig.5

library(dcurves)

library(survival)

f1<-coxph(Surv(OS.time,OS)~Pathscore+KPS+Biopsytype,surv)

f2<-coxph(Surv(OS.time,OS)~Pathscore,surv)

f3<-coxph(Surv(OS.time,OS)~KPS,surv)

f4<-coxph(Surv(OS.time,OS)~IELSG,surv)

f5<-coxph(Surv(OS.time,OS)~MSKCC,surv)

surv$nomogram_model = c(1- (summary(survfit(f1, newdata=surv), times=12)$surv))

surv$Pathscore_model = c(1- (summary(survfit(f2, newdata=surv), times=12)$surv))

surv$KPS_model = c(1- (summary(survfit(f3, newdata=surv), times=12)$surv))

surv$IELSG_model = c(1- (summary(survfit(f4, newdata=surv), times=12)$surv))

surv$MSKCC_model = c(1- (summary(survfit(f5, newdata=surv), times=12)$surv))

dca(Surv(OS.time,OS) ~ nomogram_model+Pathscore_model+KPS_model+IELSG_model+MSKCC_model,

data = surv,

time = 12,

thresholds = 1:50 / 100) %>%

plot(smooth = F)
