# Supplementary material for: Machine learning-based pathomics signature of histology slides as a novel prognostic indicator in primary central nervous system lymphoma
Source: J Neurooncol. 2024 Apr 1;168(2):283–98. doi: 10.1007/s11060-024-04665-8 (PMC11147825; doi:10.1007/s11060-024-04665-8)
Supplement: Supplementary file 3 — Supplementary file3 (DOCX 41987 KB) [file 11060_2024_4665_MOESM3_ESM.docx]

**Article title:** Machine learning-based pathomics signature of histology slides as a novel prognostic indicator in primary central nervous system lymphoma

**Journal name:** Journal of Neuro-Oncology

**Author names:** Ling Duan^1^, Yongqi He^1^, Wenhui Guo^1^, Yanru Du^2^, Shuo Yin^1^, Shoubo Yang^1^, Gehong Dong^2*^, Wenbin Li^1*^, Feng Chen^1*^

**Affiliations:** 1. Department of Neuro-Oncology, Cancer Center, Beijing Tiantan Hospital, Capital Medical University, Beijing, 100070, China; 2. Department of Pathology, Beijing Tiantan Hospital, Capital Medical University, Beijing, 100070, China

**Corresponding author:** Gehong Dong, E-mail: 13520157603@126.com; Wenbin Li, Email: liwenbin@ccmu.edu.cn; Feng Chen, Email: chenfeng406@sina.com.


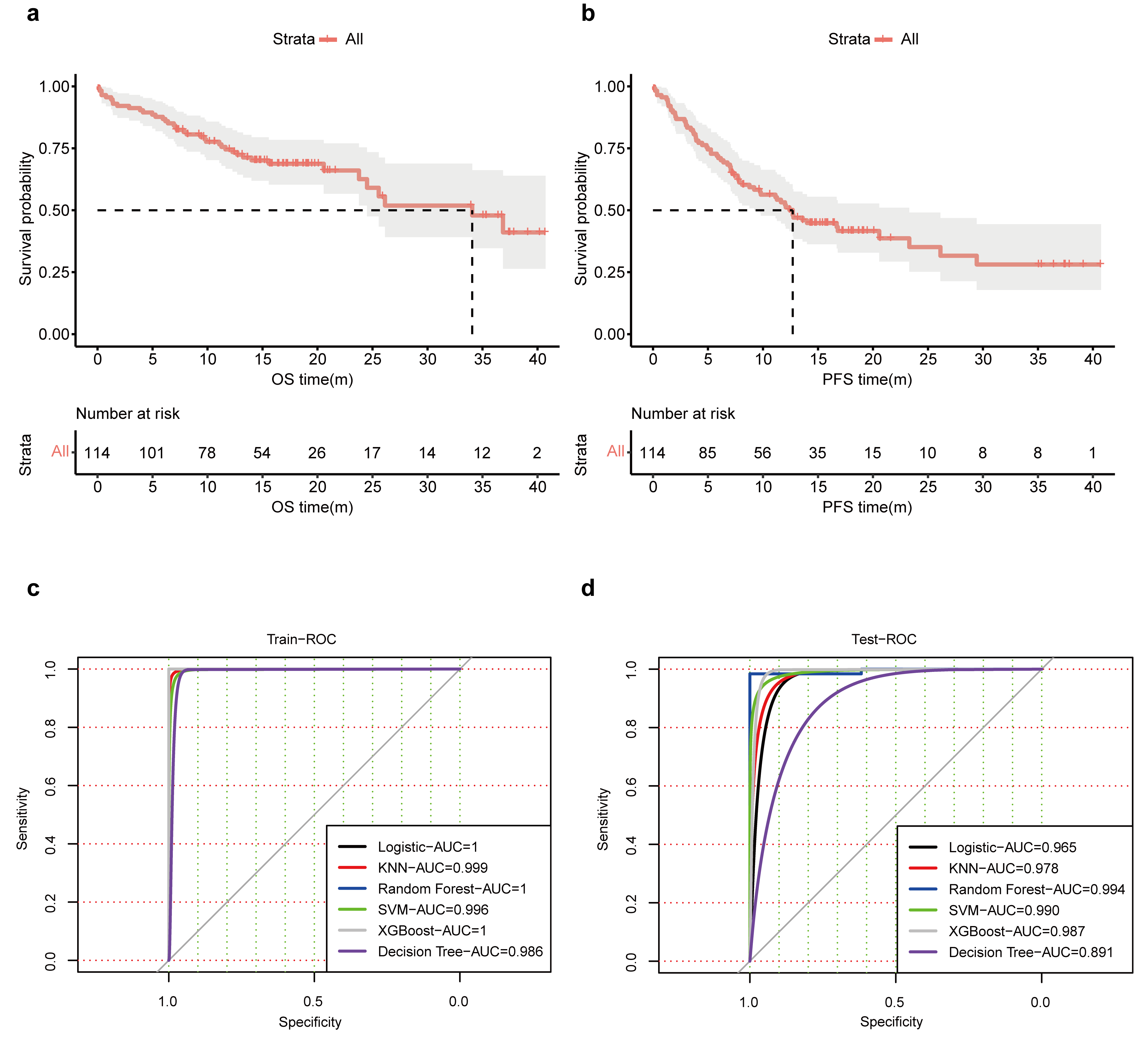


**Supplemental Fig.1 Kaplan-Meier survival analysis of combined cohorts and classifiers performance for distinguishing tumor from adjacent normal tissues. a.** The overall survival (OS) curve in the entire cohort. The median OS is 34.07 months (95%CI: 24.50-Not Reached (NR)). **b.** The progression-free survival (PFS) curve in the entire cohort. The median PFS is 12.70 months (95%CI: 9.20-23.30). **c.** Receiver operator characteristics (ROC) curves of six machine-learning classifiers of distinguishing tumors from adjacent normal tissues in the training cohort. **d.** ROC curves of six machine-learning classifiers of distinguishing tumors from adjacent normal tissues in the testing cohort.


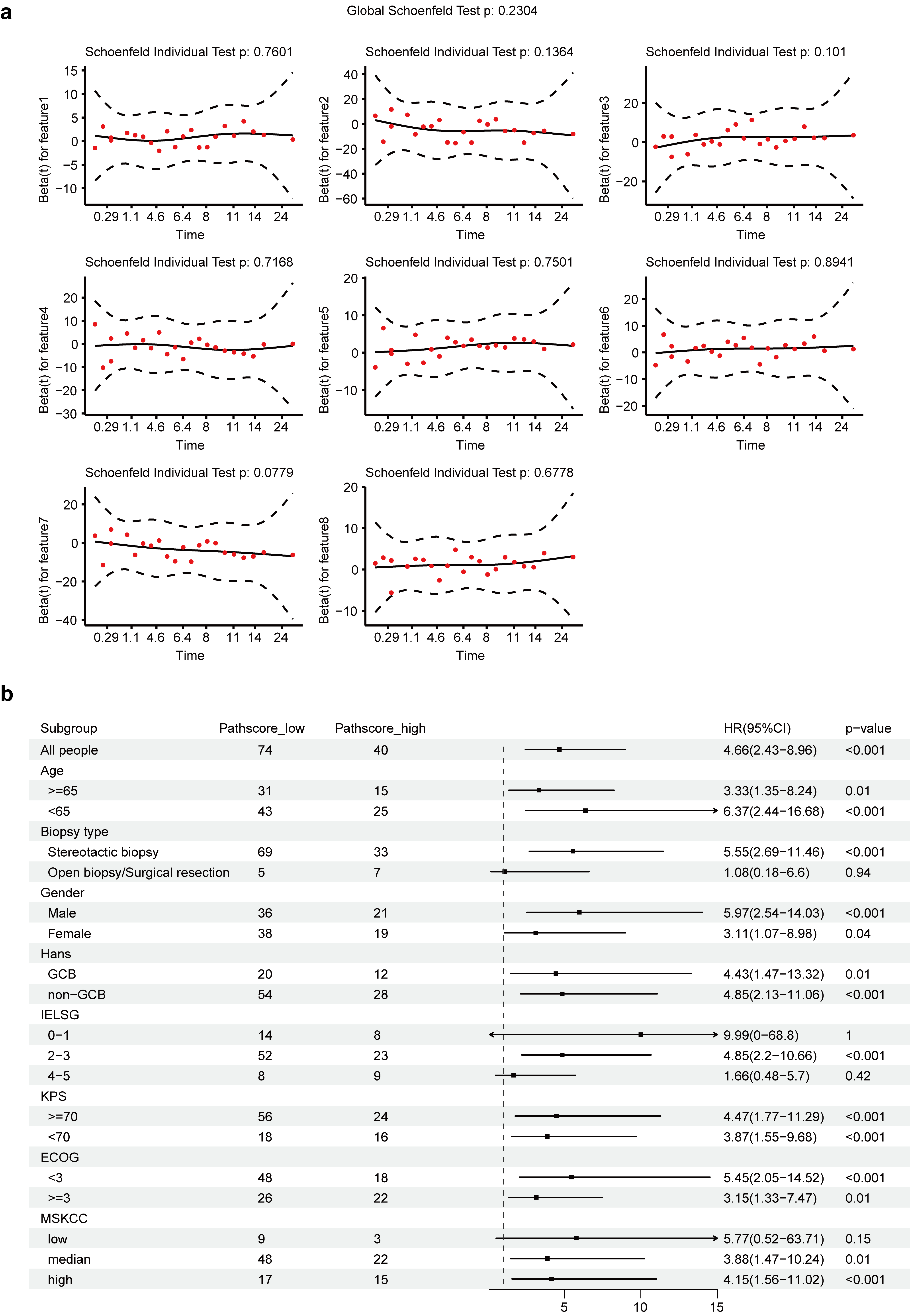


**Supplemental Fig.2 The Schoenfeld Individual Test of the multivariate Cox regression model and forest plot of pathomics score for the combined cohort. a.** The Schoenfeld Individual Test of eight selected features (Global Schoenfeld Test, p = 0.230; each covariate, p>0.05). **b.** Forest plots revealed the prognostic risk of Path-score in different subgroups.


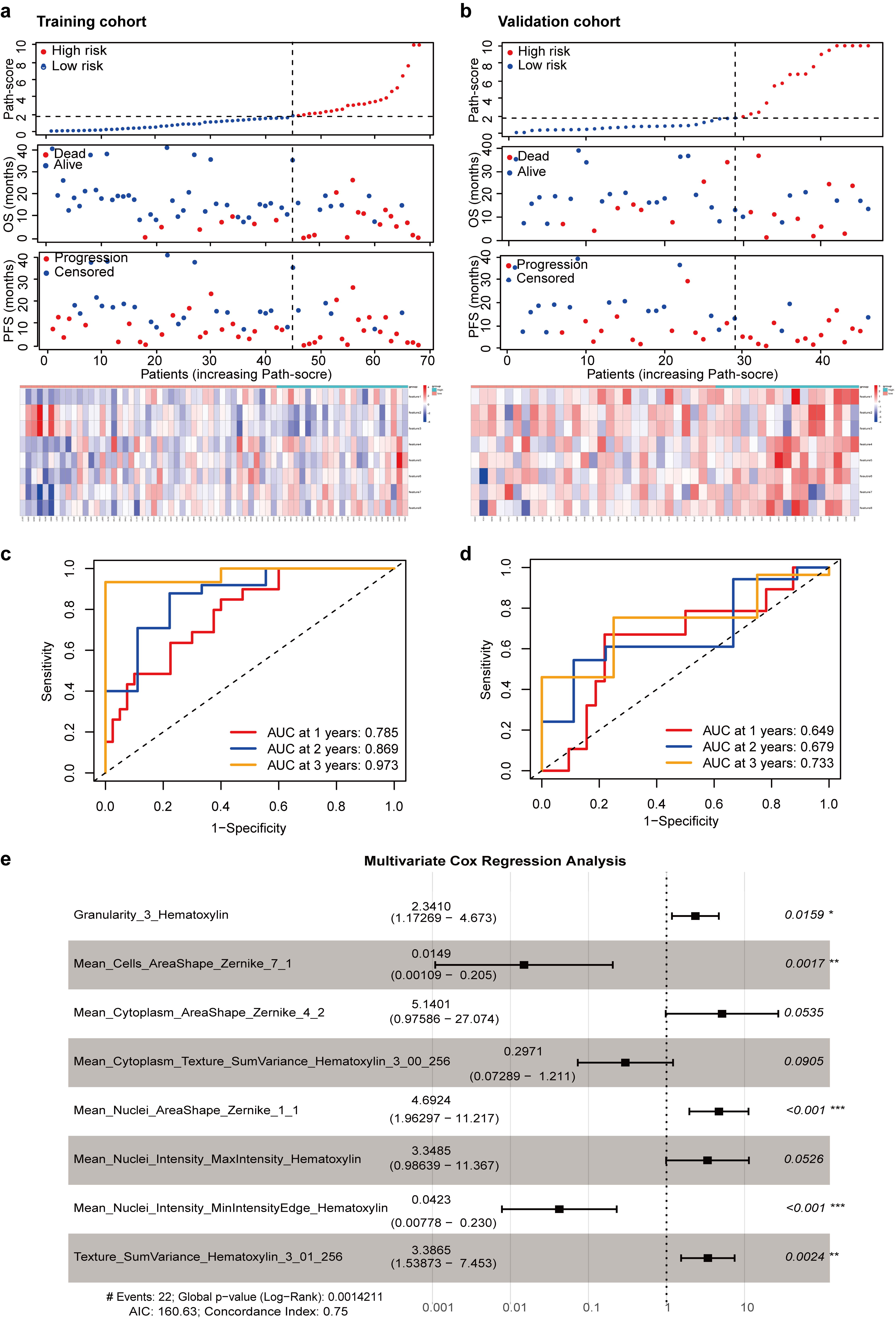


**Supplementary Fig.3 Prognostic analysis of the pathomics signature.** **a.** The distribution of the Path-score, the corresponding survival status, and eight features in the training cohort. High Path-score was associated with a higher risk of death. **b.** The distribution of the Path-score, the corresponding survival status, and eight features in the validation cohort. High Path-score was associated with a higher risk of progression. **c.** Time-dependent ROC curves demonstrated that during the 1-, 2-, and 3-year follow-ups, the AUC values were 0.785 (95%CI: 0.668-0.902), 0.869 (95%CI: 0.730-1.000), and 0.973 (0.927-1.000) in the training cohort, respectively. **d.** The AUC for 1-, 2-, and 3-year OS were 0.649 (95% CI: 0.443-0.855), 0.679 (95%CI: 0.445-0.913), and 0.733 (95%CI: 0.506-0.960) in the validation cohort, respectively. **e.** Five among the final eight features revealed independent prognostic value in multivariate Cox regression analysis (p < 0.05).


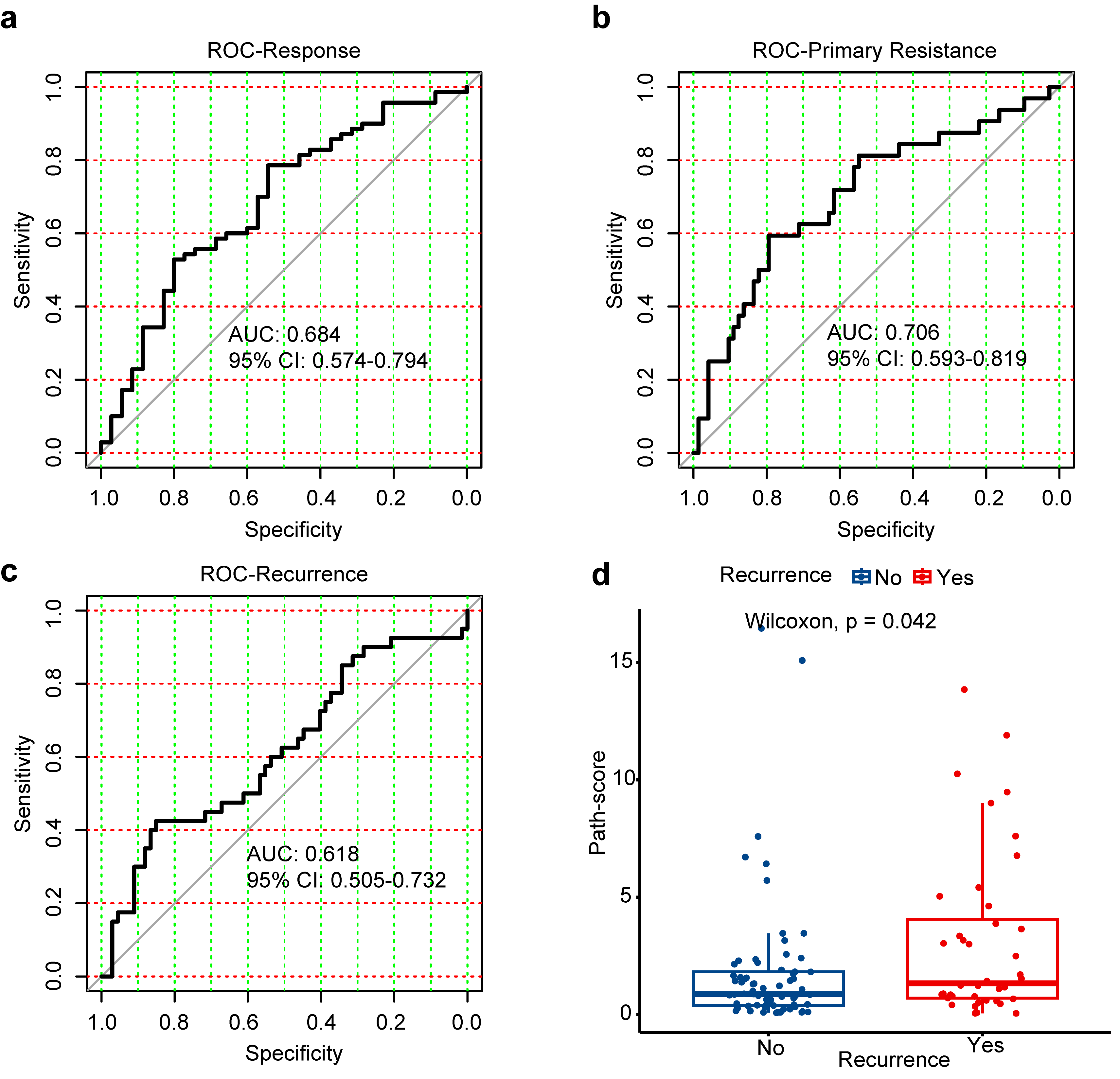


**Supplementary Fig.4** **The performance of Path-score predicting treatment response, primary tumor resistance, and recurrence.** **a.** ROC curves for distinguishing Responders from patients in the entire cohort. **b.** ROC curves for distinguishing primary resistance from patients in the entire cohort. **c.** ROC curves for predicting patients' recurrence. **d.** The distribution of Path-score based on the recurrence in the entire cohort. p = 0.042 by the Wilcoxon test.


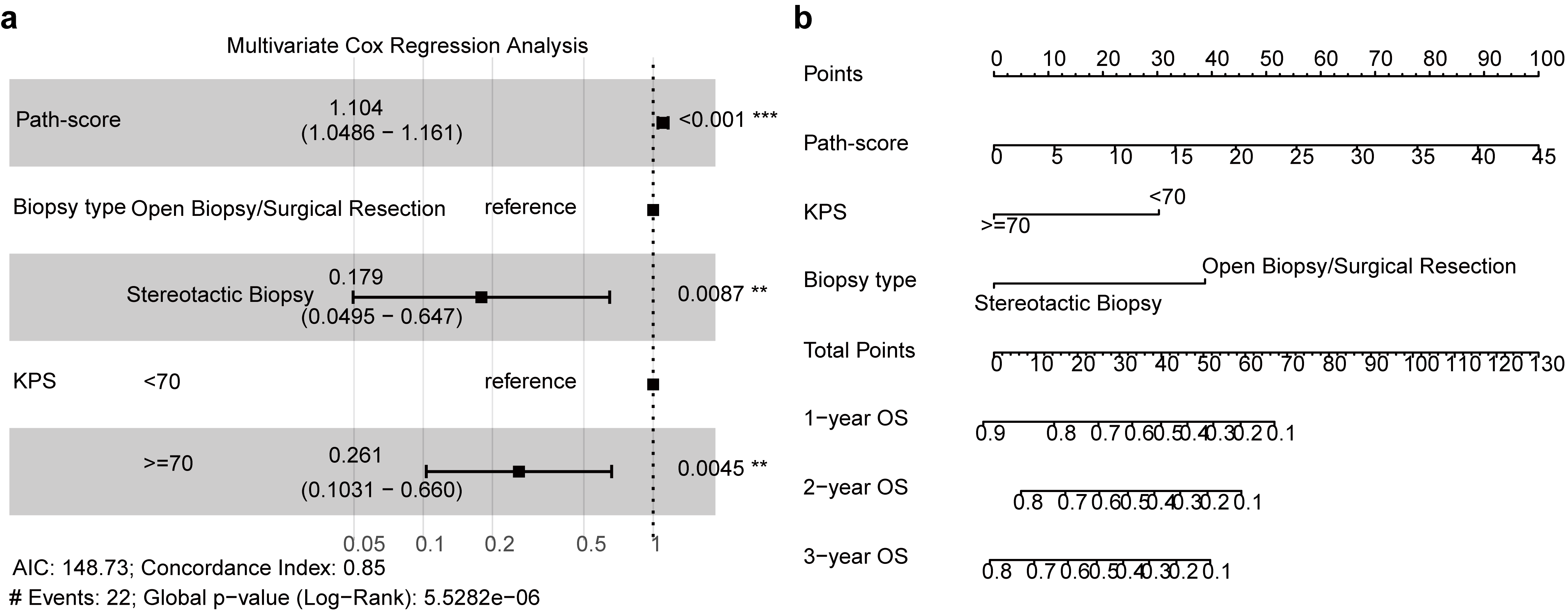


**Supplementary Fig.5 Development of the pathomics nomogram for the prediction of overall survival. a.** The backward stepwise multivariate Cox regression analysis showed that the Path-score, Biopsy type, and KPS were independent predictors of overall survival (OS). (p < 0.05). **b.** Nomogram based on Path-score and clinicopathologic factors for 1-, 2- and 3-year OS prediction of PCNSL patients.


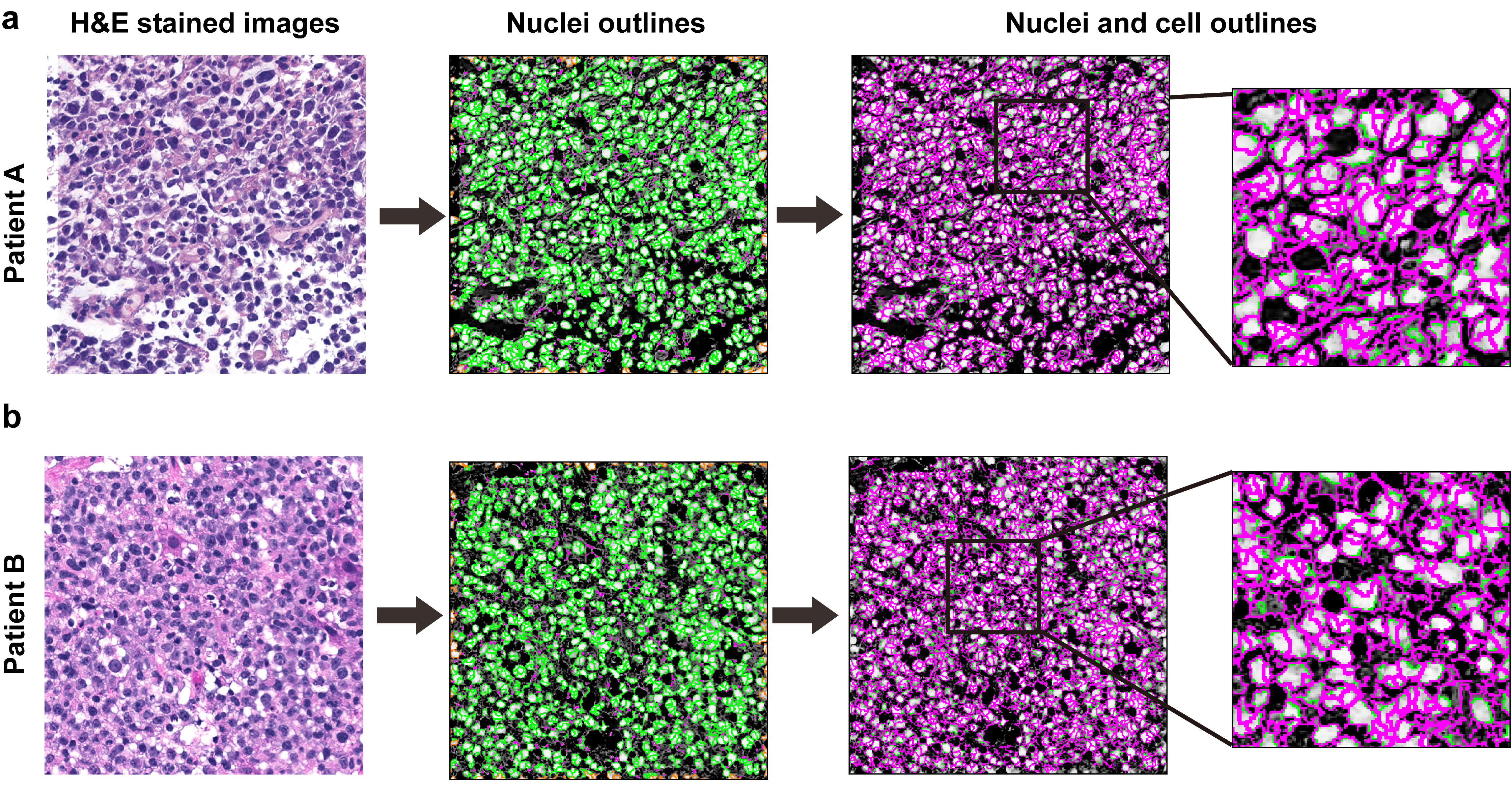


**Supplementary Fig.6 Representative H&E stained and processed images of two PCNSL patients with different survival outcomes.** **a.** Sample image of PCNSL with long survival. This patient suffered from non-GCB PCNSL and survived more than 40 months after diagnosis. **b.** Sample image of PCNSL with short survival. This patient suffered from non-GCB PCNSL and survived less than 5.5 months after diagnosis.
