# Supplementary material for: Machine learning-based pathomics signature of histology slides as a novel prognostic indicator in primary central nervous system lymphoma
Source: J Neurooncol. 2024 Apr 1;168(2):283–98. doi: 10.1007/s11060-024-04665-8 (PMC11147825; doi:10.1007/s11060-024-04665-8)
Supplement: Supplementary file 4 — Supplementary file4 (DOCX 84 KB) [file 11060_2024_4665_MOESM4_ESM.docx]

**Article title:** Machine learning-based pathomics signature of histology slides as a novel prognostic indicator in primary central nervous system lymphoma

**Journal name:** Journal of Neuro-Oncology

**Author names:** Ling Duan^1^, Yongqi He^1^, Wenhui Guo^1^, Yanru Du^2^, Shuo Yin^1^, Shoubo Yang^1^, Gehong Dong^2*^, Wenbin Li^1*^, Feng Chen^1*^

**Affiliations:** 1. Department of Neuro-Oncology, Cancer Center, Beijing Tiantan Hospital, Capital Medical University, Beijing, 100070, China; 2. Department of Pathology, Beijing Tiantan Hospital, Capital Medical University, Beijing, 100070, China

**Corresponding author:** Gehong Dong, E-mail: 13520157603@126.com; Wenbin Li, Email: liwenbin@ccmu.edu.cn; Feng Chen, Email: chenfeng406@sina.com.

**Supplementary Table S1.** **Summary of the pathomics features**

| Features category | Cellprofiler module | Feature name |
| --- | --- | --- |
| Image Quality | Measure Image Quality | FocusScore_H/E  LocalFocusScore_H/E  Correlation_H/E  PowerLogLogSlope_H/E  ThresholdOtsu_H/E_2W |
| Image Intensity | Measure Image Intensity | LowerQuartileIntensity_H/E  MADIntensity_H/E  MaxIntensity_H/E  MeanIntensity_H/E  MedianIntensity_H/E  PercentMaximal_H/E  StdIntensity_H/E  UpperQuartileIntensity_H/E |
| Image Granularity | Measure Granularity | Granularity_n_H/E (from 1 to 16) |
| Image colocalization | Measure Colocalization | Correlation_Eosin_Haematoxylin  Costes_Eosin_Haematoxylin  Costes_Haematoxylin_Eosin  Manders_Eosin_Haematoxylin  Manders_Haematoxylin_Eosin  Overlap_Eosin_Haematoxylin  RWC_Eosin_Haematoxylin  RWC_Haematoxylin_Eosin  Slope_Eosin_Haematoxylin |
| Object Intensity | Measure Object Intensity  (Mean_Cells/Cytoplasm/Nuclei) | Intensity_IntegratedIntensityEdge_H/E  Intensity_IntegratedIntensity_H/E  Intensity_LowerQuartileIntensity_H/E  Intensity_MADIntensity_H/E  Intensity_MassDisplacement_H/E  Intensity_MaxIntensityEdge_H/E  Intensity_MaxIntensity_H/E  Intensity_MeanIntensityEdge_H/E  Intensity_MeanIntensity_H/E  Intensity_MedianIntensity_H/E  Intensity_MinIntensityEdge_H/E  Intensity_MinIntensity_H/E  Intensity_StdIntensityEdge_H/E  Intensity_StdIntensity_H/E  Intensity_UpperQuartileIntensity_H/E  Location_CenterMassIntensity_X_H/E  Location_CenterMassIntensity_Y_H/E  Location_Center_X  Location_Center_Y  Location_MaxIntensity_X_H/E  Location_MaxIntensity_Y_H/E |
| Object Neighbors | Measure Object Neighbors | Mean_Cells_Parent_Nuclei  Mean_Cytoplasm_Parent_Cells  Mean_Cytoplasm_Parent_Nuclei  Mean_Nuclei_Neighbors_AngleBetweenNeighbors_Cytoplasm_Expanded  Mean_Nuclei_Neighbors_FirstClosestDistance_Cytoplasm_Expanded  Mean_Nuclei_Neighbors_FirstClosestObjectNumber_Cytoplasm_Expanded  Mean_Nuclei_Neighbors_NumberOfNeighbors_Cytoplasm_Expanded  Mean_Nuclei_Neighbors_PercentTouching_Cytoplasm_Expanded  Mean_Nuclei_Neighbors_SecondClosestDistance_Cytoplasm_Expanded  Mean_Nuclei_Neighbors_SecondClosestObjectNumber_Cytoplasm_Expanded |
| Object Size Shape | Measure Object Size Shape  (Mean_Cells/Cytoplasm/Nuclei) | AreaShape _Area  AreaShape _ BoundingBoxArea  AreaShape _BoundingBoxMaximum_X  AreaShape_BoundingBoxMaximum_Y  AreaShape_BoundingBoxMinimum_X  AreaShape_BoundingBoxMinimum_Y  AreaShape_Center_X  AreaShape_Center_Y  AreaShape_Compactness  AreaShape_ConvexArea  AreaShape_Eccentricity  AreaShape_EquivalentDiameter  AreaShape_EulerNumber  AreaShape_Extent  AreaShape_FormFactor  AreaShape_MajorAxisLength  AreaShape_MaxFeretDiameter  AreaShape_MaximumRadius  AreaShape_MeanRadius  AreaShape_MedianRadius  AreaShape_MinFeretDiameter  AreaShape_MinorAxisLength  AreaShape_Orientation  AreaShape_Perimeter  AreaShape_Solidity  AreaShape_Zernike_0_0 30  (Zernike polynomials, from 0 to 9) |
| Object Texture  (Haralick features) | Measure Texture  (Mean_Cells/Cytoplasm/Nuclei) | Texture_AngularSecondMoment_H/E_3_00/01/02/03_256  Texture_Contrast_H/E _3_00/01/02/03_256  Texture_Correlation_H/E _3_00/01/02/03_256  Texture_DifferenceEntropy_H/E _3_00/01/02/03_256  Texture_DifferenceVariance_H/E _3_00/01/02/03_256  Texture_Entropy_H/E _3_00/01/02/03_256  Texture_InfoMeas1_H/E _3_00/01/02/03_256  Texture_InfoMeas2_H/E _3_00/01/02/03_256  Texture_InverseDifferenceMoment_H/E _3_00/01/02/03_256  Texture_SumAverage_H/E _3_00/01/02/03_256  Texture_SumEntropy_H/E_3_00/01/02/03_256  Texture_SumVariance_H/E _3_00/01/02/03_256  Texture_Variance_H/E_3_00/01/02/03_256 |
| Image Texture  (Haralick features) | Measure Texture | AngularSecondMoment_H/E_3_00/01/02/03_256  Contrast_H/E_3_00/01/02/03_256  Correlation_H/E_3_00/01/02/03_256  DifferenceEntropy_H/E_3_00/01/02/03_256  DifferenceVariance_H/E_3_00/01/02/03_256  Entropy_H/E_3_00/01/02/03_256  InfoMeas1_H/E_3_00/01/02/03_256  InfoMeas2_H/E_3_00/01/02/03_256  InverseDifferenceMoment_H/E_3_00/01/02/03_256  SumAverage_H/E_3_00/01/02/03_256  SumEntropy_H/E_3_00/01/02/03_256  SumVariance_H/E_3_00/01/02/03_256  Variance_H/E_3_00/01/02/03_256  Threshold_FinalThreshold_Cells/Nuclei  Threshold_OrigThreshold_Cells/Nuclei  Threshold_SumOfEntropies_Cells/Nuclei  Threshold_WeightedVariance_Cells/Nuclei |

**Supplementary Table S2. Characteristics of patients in the training and validation cohorts**

| **Characteristics** | **Combined cohort** | **Validation cohort** | **Training cohort** | **p-value** |
| --- | --- | --- | --- | --- |
|  | **N=114** | **N=46** | **N=68** |  |
| Age |  |  |  | 0.234 |
| <65 | 68 (59.65%) | 31 (67.39%) | 37 (54.41%) |  |
| >=65 | 46 (40.35%) | 15 (32.61%) | 31 (45.59%) |  |
| Sex |  |  |  | 0.567 |
| Female | 57 (50.00%) | 25 (54.35%) | 32 (47.06%) |  |
| Male | 57 (50.00%) | 21 (45.65%) | 36 (52.94%) |  |
| Biopsy type |  |  |  | 0.064 |
| Surgical resection  /Open biopsy | 12 (10.53%) | 8 (17.39%) | 4 (5.88%) |  |
| Stereotactic biopsy | 102 (89.47%) | 38 (82.61%) | 64 (94.12%) |  |
| Hans |  |  |  | 0.548 |
| GCB | 32 (28.07%) | 11 (23.91%) | 21 (30.88%) |  |
| non-GCB | 82 (71.93%) | 35 (76.09%) | 47 (69.12%) |  |
| Tumor Size |  |  |  | 0.594 |
| <5cm | 86 (75.44%) | 33 (71.74%) | 53 (77.94%) |  |
| >=5cm | 28 (24.56%) | 13 (28.26%) | 15 (22.06%) |  |
| Deep Lesions |  |  |  | 1.000 |
| Absent | 29 (25.44%) | 12 (26.09%) | 17 (25.00%) |  |
| Present | 85 (74.56%) | 34 (73.91%) | 51 (75.00%) |  |
| Number of lesions |  |  |  | 0.857 |
| Single | 47 (41.23%) | 18 (39.13%) | 29 (42.65%) |  |
| Multiple | 67 (58.77%) | 28 (60.87%) | 39 (57.35%) |  |
| KPS |  |  |  | 0.029 |
| <70 | 34 (29.83%) | 8 (17.39%) | 26 (38.24%) |  |
| >=70 | 80 (70.17%) | 38 (82.61%) | 42 (61.76%) |  |
| ECOG |  |  |  | 0.135 |
| <3 | 66 (57.90%) | 31 (67.39%) | 35 (51.47%) |  |
| >=3 | 48 (42.10%) | 15 (32.61%) | 33 (48.53%) |  |
| IELSG |  |  |  | 0.299 |
| 0-1 | 22 (19.30%) | 9 (19.57%) | 13 (19.12%) |  |
| 2-3 | 75 (65.79%) | 33 (71.74%) | 42 (61.77%) |  |
| 4-5 | 17 (14.91%) | 4 (8.69%) | 13 (19.11%) |  |
| MSKCC |  |  |  | 0.041 |
| low | 12 (10.53%) | 6 (13.04%) | 6 (8.82%) |  |
| intermediate | 70 (61.40%) | 33 (71.74%) | 37 (54.41%) |  |
| high | 32 (28.07%) | 7 (15.22%) | 25 (36.77%) |  |
| Radiotherapy |  |  |  | 0.097 |
| No | 100 (87.72%) | 37 (80.43%) | 63 (92.65%) |  |
| Yes | 14 (12.28%) | 9 (19.57%) | 5 (7.35%) |  |
| Treatment |  |  |  | 0.040 |
| CT | 91 (79.83%) | 36 (78.26%) | 55 (80.88%) |  |
| CT+RT | 14 (12.28%) | 9 (19.57%) | 5 (7.35%) |  |
| Supportive | 9 (7.89%) | 1 (2.17%) | 8 (11.77%) |  |
| BTKi |  |  |  | 0.827 |
| No | 72 (63.16%) | 28 (60.87%) | 44 (64.71%) |  |
| Yes | 42 (36.84%) | 18 (39.13%) | 24 (35.29%) |  |
| Consolidation |  |  |  | 1.000 |
| No | 82 (71.93%) | 33 (71.74%) | 49 (72.06%) |  |
| Yes | 32 (28.07%) | 13 (28.26%) | 19 (27.94%) |  |
| Treatment Evaluation |  |  |  | 0.386 |
| CR | 61 (58.10%) | 23 (51.11%) | 38 (63.33%) |  |
| PD | 32 (30.48%) | 15 (33.33%) | 17 (28.33%) |  |
| PR | 9 (8.57%) | 6 (13.34%) | 3 (5.01%) |  |
| SD | 3 (2.85%) | 1 (2.22%) | 2 (3.33%) |  |
| Response |  |  |  | 0.834 |
| Non-Responders | 35 (33.33%) | 16 (35.56%) | 19 (31.67%) |  |
| Responders | 70 (66.67%) | 29 (64.44%) | 41 (68.33%) |  |
| Primary Resistance |  |  |  | 0.879 |
| No | 72 (68.57%) | 30 (66.67%) | 42 (70.00%) |  |
| Yes | 33 (31.43%) | 15 (33.33%) | 18 (30.00%) |  |

**Supplementary Tabel S3.** **Performance of Six Machine-Learning Classifiers**

| Classifiers | Accuracy | AUC-Train (95%CI) | AUC-Test (95%CI) | F1 |
| --- | --- | --- | --- | --- |
| Logistic | 0.923 | 1.000 (1.000-1.000) | 0.965 (0.917-0.996) | 0.919 |
| KNN | 0.923 | 0.999 (0.998-1.000) | 0.978 (0.964-0.999) | 0.917 |
| XGBoost | 0.957 | 1.000 (1.000-1.000) | 0.987 (0.982-1.000) | 0.961 |
| SVM | 0.949 | 0.996 (0.995-1.000) | 0.990 (0.982-1.000) | 0.950 |
| Decision Tree | 0.838 | 0.986 (0.965-1.000) | 0.891 (0.771-0.913) | 0.843 |
| Random Forest | 0.974 | 1.000 (1.000-1.000) | 0.994 (0.982-1.000) | 0.976 |

**Supplemental Table S4: The top 30 important features selected by Random Forest**

| Features | Mean Decrease Accuracy |
| --- | --- |
| Granularity_3_Eosin | 6.049 |
| Granularity_2_Eosin | 5.587 |
| Granularity_1_Eosin | 4.645 |
| Mean_Cytoplasm_Texture_InfoMeas1_Hematoxylin_3_03_256 | 4.645 |
| Correlation_Correlation_Eosin_Hematoxylin | 4.614 |
| ImageQuality_Correlation_Hematoxylin_20 | 4.199 |
| Mean_Nuclei_Intensity_MADIntensity_Hematoxylin | 4.079 |
| Texture_AngularSecondMoment_Eosin_3_02_256 | 4.055 |
| Mean_Cells_Intensity_IntegratedIntensityEdge_Hematoxylin | 4.053 |
| Mean_Nuclei_Texture_InfoMeas1_Eosin_3_03_256 | 4.023 |
| Correlation_Overlap_Eosin_Hematoxylin | 3.869 |
| Texture_AngularSecondMoment_Eosin_3_00_256 | 3.869 |
| ImageQuality_MeanIntensity_Eosin | 3.837 |
| Texture_AngularSecondMoment_Eosin_3_03_256 | 3.814 |
| ImageQuality_ThresholdOtsu_Eosin_2W | 3.799 |
| Mean_Nuclei_Intensity_MeanIntensity_Hematoxylin | 3.783 |
| Mean_Cytoplasm_Texture_InfoMeas1_Hematoxylin_3_01_256 | 3.733 |
| Mean_Cytoplasm_AreaShape_Compactness | 3.662 |
| Intensity_MeanIntensity_Eosin | 3.639 |
| Correlation_Costes_Hematoxylin_Eosin | 3.625 |
| Mean_Cells_AreaShape_Zernike_1_1 | 3.608 |
| Threshold_WeightedVariance_Nuclei | 3.571 |
| Mean_Cytoplasm_Texture_InfoMeas1_Hematoxylin_3_00_256 | 3.518 |
| Mean_Cells_AreaShape_Perimeter | 3.513 |
| Mean_Cells_AreaShape_Compactness | 3.468 |
| Mean_Nuclei_Texture_InfoMeas1_Eosin_3_01_256 | 3.441 |
| Texture_AngularSecondMoment_Eosin_3_01_256 | 3.431 |
| Mean_Cells_Texture_Correlation_Eosin_3_01_256 | 3.425 |
| Granularity_2_Hematoxylin | 3.391 |
| ImageQuality_LocalFocusScore_Hematoxylin_20 | 3.367 |

**Supplemental Table S5: The significant features in the univariate Cox regression analysis**

| Features | HR | 95%CI | p-value |
| --- | --- | --- | --- |
| Granularity_12_Hematoxylin | 0.563 | 0.319-0.991 | 0.047 |
| Granularity_2_Hematoxylin | 1.378 | 1.047-1.814 | 0.022 |
| Granularity_3_Hematoxylin | 1.415 | 1.010-1.982 | 0.044 |
| ImageQuality_FocusScore_Hematoxylin | 1.362 | 1.015-1.830 | 0.040 |
| Mean_Cells_AreaShape_Zernike_3_1 | 1.533 | 1.064-2.210 | 0.022 |
| Mean_Cells_AreaShape_Zernike_4_2 | 0.677 | 0.503-0.910 | 0.010 |
| Mean_Cells_AreaShape_Zernike_5_3 | 0.701 | 0.507-0.971 | 0.033 |
| Mean_Cells_AreaShape_Zernike_6_4 | 0.680 | 0.485-0.953 | 0.025 |
| Mean_Cells_AreaShape_Zernike_7_1 | 0.690 | 0.481-0.990 | 0.044 |
| Mean_Cells_AreaShape_Zernike_7_3 | 0.666 | 0.467-0.951 | 0.025 |
| Mean_Cells_AreaShape_Zernike_7_5 | 0.694 | 0.494-0.976 | 0.036 |
| Mean_Cells_AreaShape_Zernike_8_6 | 0.714 | 0.513-0.993 | 0.046 |
| Mean_Cells_AreaShape_Zernike_9_3 | 0.696 | 0.496-0.978 | 0.037 |
| Mean_Cells_AreaShape_Zernike_9_5 | 0.696 | 0.494-0.981 | 0.038 |
| Mean_Cells_AreaShape_Zernike_9_7 | 0.706 | 0.505-0.987 | 0.042 |
| Mean_Cells_Intensity_MaxIntensityEdge_Hematoxylin | 1.670 | 1.174-2.374 | 0.004 |
| Mean_Cells_Intensity_MaxIntensity_Hematoxylin | 1.571 | 1.138-2.169 | 0.006 |
| Mean_Cells_Intensity_MeanIntensity_Hematoxylin | 1.546 | 1.042-2.293 | 0.031 |
| Mean_Cells_Intensity_MedianIntensity_Hematoxylin | 1.496 | 1.022-2.190 | 0.038 |
| Mean_Cells_Intensity_UpperQuartileIntensity_Hematoxylin | 1.647 | 1.141-2.378 | 0.008 |
| Mean_Cells_Texture_Correlation_Hematoxylin_3_00_256 | 1.426 | 1.016-2.002 | 0.040 |
| Mean_Cells_Texture_DifferenceVariance_Hematoxylin_3_00_256 | 0.666 | 0.467-0.952 | 0.026 |
| Mean_Cells_Texture_DifferenceVariance_Hematoxylin_3_01_256 | 0.658 | 0.459-0.943 | 0.023 |
| Mean_Cells_Texture_DifferenceVariance_Hematoxylin_3_02_256 | 0.687 | 0.480-0.983 | 0.040 |
| Mean_Cells_Texture_DifferenceVariance_Hematoxylin_3_03_256 | 0.684 | 0.473-0.988 | 0.043 |
| Mean_Cells_Texture_InverseDifferenceMoment_Hematoxylin_3_00_256 | 1.517 | 1.040-2.213 | 0.031 |
| Mean_Cells_Texture_InverseDifferenceMoment_Hematoxylin_3_01_256 | 1.456 | 1.002-2.114 | 0.049 |
| Mean_Cells_Texture_InverseDifferenceMoment_Hematoxylin_3_02_256 | 1.465 | 1.011-2.123 | 0.044 |
| Mean_Cells_Texture_SumAverage_Hematoxylin_3_00_256 | 1.624 | 1.084-2.432 | 0.019 |
| Mean_Cells_Texture_SumAverage_Hematoxylin_3_01_256 | 1.693 | 1.119-2.559 | 0.013 |
| Mean_Cells_Texture_SumAverage_Hematoxylin_3_02_256 | 1.606 | 1.073-2.404 | 0.021 |
| Mean_Cells_Texture_SumAverage_Hematoxylin_3_03_256 | 1.653 | 1.095-2.497 | 0.017 |
| Mean_Cytoplasm_AreaShape_Zernike_4_2 | 0.661 | 0.470-0.928 | 0.017 |
| Mean_Cytoplasm_AreaShape_Zernike_5_3 | 0.715 | 0.525-0.974 | 0.034 |
| Mean_Cytoplasm_AreaShape_Zernike_6_4 | 0.705 | 0.511-0.974 | 0.034 |
| Mean_Cytoplasm_AreaShape_Zernike_7_5 | 0.707 | 0.511-0.976 | 0.035 |
| Mean_Cytoplasm_AreaShape_Zernike_8_6 | 0.724 | 0.525-0.999 | 0.049 |
| Mean_Cytoplasm_AreaShape_Zernike_9_5 | 0.721 | 0.524-0.992 | 0.045 |
| Mean_Cytoplasm_AreaShape_Zernike_9_7 | 0.709 | 0.512-0.982 | 0.038 |
| Mean_Cytoplasm_Intensity_IntegratedIntensityEdge_Hematoxylin | 1.368 | 1.006-1.861 | 0.046 |
| Mean_Cytoplasm_Intensity_MaxIntensityEdge_Hematoxylin | 1.597 | 1.156-2.206 | 0.005 |
| Mean_Cytoplasm_Intensity_MaxIntensity_Hematoxylin | 1.564 | 1.141-2.144 | 0.005 |
| Mean_Cytoplasm_Intensity_MeanIntensityEdge_Hematoxylin | 1.520 | 1.026-2.252 | 0.037 |
| Mean_Cytoplasm_Intensity_MeanIntensity_Hematoxylin | 1.482 | 1.008-2.180 | 0.045 |
| Mean_Cytoplasm_Intensity_UpperQuartileIntensity_Hematoxylin | 1.522 | 1.055-2.193 | 0.025 |
| Mean_Cytoplasm_Texture_Correlation_Hematoxylin_3_00_256 | 1.511 | 1.033-2.211 | 0.034 |
| Mean_Cytoplasm_Texture_Correlation_Hematoxylin_3_02_256 | 1.527 | 1.041-2.240 | 0.030 |
| Mean_Cytoplasm_Texture_DifferenceVariance_Hematoxylin_3_00_256 | 0.664 | 0.477-0.924 | 0.015 |
| Mean_Cytoplasm_Texture_DifferenceVariance_Hematoxylin_3_01_256 | 0.667 | 0.479-0.927 | 0.016 |
| Mean_Cytoplasm_Texture_DifferenceVariance_Hematoxylin_3_02_256 | 0.680 | 0.488-0.948 | 0.023 |
| Mean_Cytoplasm_Texture_DifferenceVariance_Hematoxylin_3_03_256 | 0.683 | 0.488-0.956 | 0.026 |
| Mean_Cytoplasm_Texture_SumAverage_Hematoxylin_3_01_256 | 1.512 | 1.022-2.238 | 0.039 |
| Mean_Cytoplasm_Texture_SumAverage_Hematoxylin_3_03_256 | 1.509 | 1.019-2.235 | 0.040 |
| Mean_Cytoplasm_Texture_SumVariance_Hematoxylin_3_00_256 | 1.565 | 1.155-2.120 | 0.004 |
| Mean_Cytoplasm_Texture_SumVariance_Hematoxylin_3_01_256 | 1.450 | 1.085-1.938 | 0.012 |
| Mean_Cytoplasm_Texture_SumVariance_Hematoxylin_3_02_256 | 1.570 | 1.161-2.122 | 0.003 |
| Mean_Cytoplasm_Texture_SumVariance_Hematoxylin_3_03_256 | 1.440 | 1.072-1.935 | 0.016 |
| Mean_Cytoplasm_Texture_Variance_Hematoxylin_3_00_256 | 1.356 | 1.003-1.832 | 0.048 |
| Mean_Cytoplasm_Texture_Variance_Hematoxylin_3_01_256 | 1.393 | 1.036-1.873 | 0.028 |
| Mean_Cytoplasm_Texture_Variance_Hematoxylin_3_02_256 | 1.375 | 1.016-1.860 | 0.039 |
| Mean_Cytoplasm_Texture_Variance_Hematoxylin_3_03_256 | 1.400 | 1.039-1.886 | 0.027 |
| Mean_Nuclei_AreaShape_Zernike_1_1 | 1.661 | 1.238-2.229 | 0.001 |
| Mean_Nuclei_AreaShape_Zernike_4_2 | 0.560 | 0.383-0.820 | 0.003 |
| Mean_Nuclei_AreaShape_Zernike_9_9 | 0.656 | 0.433-0.993 | 0.046 |
| Mean_Nuclei_Intensity_IntegratedIntensityEdge_Hematoxylin | 1.755 | 1.117-2.757 | 0.015 |
| Mean_Nuclei_Intensity_LowerQuartileIntensity_Hematoxylin | 1.841 | 1.194-2.839 | 0.006 |
| Mean_Nuclei_Intensity_MassDisplacement_Hematoxylin | 0.692 | 0.486-0.985 | 0.041 |
| Mean_Nuclei_Intensity_MaxIntensityEdge_Hematoxylin | 1.790 | 1.247-2.569 | 0.002 |
| Mean_Nuclei_Intensity_MaxIntensity_Hematoxylin | 1.604 | 1.154-2.228 | 0.005 |
| Mean_Nuclei_Intensity_MeanIntensityEdge_Hematoxylin | 1.840 | 1.210-2.798 | 0.004 |
| Mean_Nuclei_Intensity_MeanIntensity_Hematoxylin | 1.821 | 1.222-2.714 | 0.003 |
| Mean_Nuclei_Intensity_MedianIntensity_Hematoxylin | 1.807 | 1.234-2.644 | 0.002 |
| Mean_Nuclei_Intensity_MinIntensityEdge_Hematoxylin | 1.484 | 1.022-2.156 | 0.038 |
| Mean_Nuclei_Intensity_MinIntensity_Hematoxylin | 1.477 | 1.018-2.142 | 0.040 |
| Mean_Nuclei_Intensity_UpperQuartileIntensity_Hematoxylin | 1.765 | 1.245-2.504 | 0.001 |
| Mean_Nuclei_Neighbors_AngleBetweenNeighbors_Cytoplasm_Expanded | 1.744 | 1.220-2.493 | 0.002 |
| Mean_Nuclei_Texture_DifferenceVariance_Hematoxylin_3_00_256 | 0.601 | 0.386-0.936 | 0.024 |
| Mean_Nuclei_Texture_DifferenceVariance_Hematoxylin_3_01_256 | 0.591 | 0.379-0.922 | 0.020 |
| Mean_Nuclei_Texture_InverseDifferenceMoment_Hematoxylin_3_00_256 | 1.523 | 1.053-2.203 | 0.026 |
| Mean_Nuclei_Texture_InverseDifferenceMoment_Hematoxylin_3_01_256 | 1.472 | 1.009-2.146 | 0.045 |
| Mean_Nuclei_Texture_InverseDifferenceMoment_Hematoxylin_3_02_256 | 1.470 | 1.019-2.120 | 0.039 |
| Mean_Nuclei_Texture_InverseDifferenceMoment_Hematoxylin_3_03_256 | 1.462 | 1.004-2.128 | 0.048 |
| Mean_Nuclei_Texture_SumAverage_Hematoxylin_3_00_256 | 1.759 | 1.152-2.686 | 0.009 |
| Mean_Nuclei_Texture_SumAverage_Hematoxylin_3_01_256 | 1.789 | 1.165-2.748 | 0.008 |
| Mean_Nuclei_Texture_SumAverage_Hematoxylin_3_02_256 | 1.752 | 1.146-2.677 | 0.010 |
| Mean_Nuclei_Texture_SumAverage_Hematoxylin_3_03_256 | 1.749 | 1.142-2.681 | 0.010 |
| Texture_SumVariance_Hematoxylin_3_00_256 | 1.514 | 1.029-2.225 | 0.035 |
| Texture_SumVariance_Hematoxylin_3_01_256 | 1.542 | 1.054-2.258 | 0.026 |
| Texture_SumVariance_Hematoxylin_3_02_256 | 1.492 | 1.013-2.197 | 0.043 |
| Texture_SumVariance_Hematoxylin_3_03_256 | 1.486 | 1.010-2.188 | 0.044 |
| Threshold_WeightedVariance_Cells | 1.402 | 1.021-1.924 | 0.037 |

**Supplementary Table S6. The distribution of Path-score in subgroups.**

| Characteristics | Category | N | Median Path-score (IQR) | p-value |
| --- | --- | --- | --- | --- |
| Age | <65 | 68 | 1.145 (0.493-3.244) | 0.551 |
|  | >=65 | 46 | 1.284 (0.440-2.275) |  |
| Sex | Female | 57 | 1.122 (0.469-2.350) | 0.769 |
|  | Male | 57 | 1.321 (0.517-3.004) |  |
| Biopsy type | Stereotactic biopsy | 102 | 1.145(0.477-2.275) | 0.096 |
|  | Open biopsy  /Surgical resection | 12 | 2.937 (0.784-6.187) |  |
| Hans | Non-GCB | 82 | 1.197 (0.463-2.395) | 0.371 |
|  | GCB | 32 | 1.343 (0.632-3.215) |  |
| Tumor Size | <5cm | 86 | 1.084 (0.445-2.198) | 0.165 |
|  | >=5cm | 28 | 1.681 (0.676-3.235) |  |
| Deep Lesions | Absent | 29 | 0.859 (0.390-3.647) | 0.641 |
|  | Present | 85 | 1.246 (0.610-2.350) |  |
| Number of Lesions | Single | 47 | 1.226 (0.429-3.555) | 0.614 |
|  | Multiple | 67 | 1.236 (0.621-2.182) |  |
| Treatment Evaluation | CR | 61 | 0.858 (0.396-1.824) | **0.007** |
|  | PR | 9 | 0.775 (0.462-0.875) |  |
|  | SD | 3 | 0.859 (0.719-1.014) |  |
|  | PD | 32 | 2.313 (1.052-5.467) |  |
| Response | Non-responders | 35 | 2.150 (0.942-4.833) | **0.002** |
|  | Responders | 70 | 0.849 (0.403-1.743) |  |
| Primary Resistance | No | 73 | 0.852 (0.414-1.704) | **<0.001** |
|  | Yes | 32 | 2.313 (1.052-5.467) |  |
| KPS | <70 | 34 | 1.678 (0.955-2.876) | **0.028** |
|  | >=70 | 80 | 0.867 (0.411-2.450) |  |
| ECOG | <3 | 66 | 0.883 (0.403-1.882) | **0.037** |
|  | >=3 | 48 | 1.646 (0.691-3.091) |  |
| IELSG | 0-1 | 22 | 0.934 (0.352-3.074) | 0.438 |
|  | 2-3 | 75 | 1.069 (0.463-2.489) |  |
|  | 4-5 | 17 | 1.922 (1.169-2.494) |  |
| MSKCC | Low | 12 | 1.274 (0.694-1.753) | 0.151 |
|  | Intermediate | 70 | 0.867 (0.397-2.920) |  |
|  | High | 32 | 1.678 (0.876-2.621) |  |

**Supplementary Table S7. The differences between Non-Responders and Responders.**

| **Characteristics** | **Non-Responders** | **Responders** | **p-value** |
| --- | --- | --- | --- |
|  | **N=35** | **N=70** |  |
| Path-score |  |  | **0.003** |
| low | 16 (45.714%) | 54 (77.143%) |  |
| high | 19 (54.286%) | 16 (22.857%) |  |
| Age |  |  | 1.000 |
| <65 | 21 (60.000%) | 42 (60.000%) |  |
| >=65 | 14 (40.000%) | 28 (40.000%) |  |
| Sex |  |  | 0.370 |
| Female | 15 (42.857%) | 38 (54.286%) |  |
| Male | 20 (57.143%) | 32 (45.714%) |  |
| Biopsy type |  |  | 1.000 |
| Open biopsy  /Surgical resection | 3 (8.571%) | 6 (8.571%) |  |
| Stereotactic biopsy | 32 (91.429%) | 64 (91.429%) |  |
| Hans |  |  | 1.000 |
| GCB | 9 (25.714%) | 19 (27.143%) |  |
| Non-GCB | 26 (74.286%) | 51 (72.857%) |  |
| Tumor Size |  |  | 0.936 |
| <5cm | 27 (77.143%) | 52 (74.286%) |  |
| >=5cm | 8 (22.857%) | 18 (25.714%) |  |
| Deep Lesions |  |  | 0.696 |
| Absent | 8 (22.857%) | 20 (28.571%) |  |
| Present | 27 (77.143%) | 50 (71.429%) |  |
| Number of lesions |  |  | 1.000 |
| Single | 14 (40.000%) | 29 (41.429%) |  |
| Multiple | 21 (60.000%) | 41 (58.571%) |  |
| KPS |  |  | 0.236 |
| <70 | 12 (34.286%) | 15 (21.429%) |  |
| >=70 | 23 (65.714%) | 55 (78.571%) |  |
| ECOG |  |  | 0.284 |
| <3 | 19 (54.286%) | 47 (67.143%) |  |
| >=3 | 16 (45.714%) | 23 (32.857%) |  |
| IELSG |  |  | 0.175 |
| 0-1 | 4 (11.429%) | 18 (25.714%) |  |
| 2-3 | 25 (71.429%) | 45 (64.286%) |  |
| 4-5 | 6 (17.143%) | 7 (10.000%) |  |
| MSKCC |  |  | 0.435 |
| low | 3 (8.571%) | 8 (11.429%) |  |
| high | 11 (31.429%) | 14 (20.000%) |  |
| Intermediate | 21 (60.000%) | 48 (68.571%) |  |

**Supplementary Table S8. Univariate and multivariate Cox regression analyses of the Path-score and clinicopathological characteristics for overall**

**survival and progression-free survival in the training cohort**

| Characteristics | Category | Overall survival  Univariate analysis Multivariate analysis  HR (95% CI) p-value HR (95% CI) p-value | | | | Progression-free survival  Univariate analysis Multivariate analysis  HR (95% CI) p-value HR (95% CI) p-value | | | |
| --- | --- | --- | --- | --- | --- | --- | --- | --- | --- |
| Age | <65 | Reference | 0.964 |  |  | Reference | 0.787 |  |  |
|  | >=65 | 0.981 (0.423-2.274) |  |  |  | 0.918 (0.492-1.712) |  |  |  |
| Sex | Female | Reference | 0.328 |  |  | Reference | 0.920 |  |  |
|  | Male | 1.543 (0.647-3.682) |  |  |  | 0.969 (0.520-1.805) |  |  |  |
| Biopsy type | Surgical resection/Open biopsy | Reference | **0.014** | Reference | **0.003** | Reference | 0.200 |  |  |
|  | Stereotactic biopsy | 0.204 (0.058 -0.720) |  | 0.092 (0.019-0.440) |  | 0.457 (0.138-1.513) |  |  |  |
| Hans | GCB | Reference | 0.521 |  |  | Reference | 0.629 |  |  |
|  | Non-GCB | 0.752 (0.315-1.795) |  |  |  | 0.849 (0.437-1.649) |  |  |  |
| Tumor Size | <5cm | Reference | 0.381 |  |  | Reference | 0.737 |  |  |
|  | >=5cm | 1.523 (0.595-3.899) |  |  |  | 1.136 (0.540-2.391) |  |  |  |
| Deep Lesions | Absent | Reference | 0.442 |  |  | Reference | 0.806 |  |  |
|  | Present | 1.531 (0.517-4.539) |  |  |  | 1.098 (0.521-2.312) |  |  |  |
| Number of lesions | Single | Reference | 0.906 |  |  | Reference | 0.208 |  |  |
|  | Multiple | 0.950 (0.403-2.238) |  |  |  | 0.662 (0.349-1.258) |  |  |  |
| KPS | <70 | Reference | **<0.001** | Reference | **0.006** | Reference | 0.092 |  |  |
|  | >=70 | 0.214 (0.087-0.527) |  | 0.084 (0.014-0.492) |  | 0.585 (0.313-1.091) |  |  |  |
| ECOG | <3 | Reference | **0.006** | Reference | 0.210 | Reference | **0.040** | Reference | 0.367 |
|  | >=3 | 3.766 (1.465-9.678) |  | 0.313 (0.051-1.926) |  | 1.936 (1.032-3.632) |  | 1.404 (0.671-2.934) |  |
| IELSG | 0-1 | Reference | - | Reference | - | Reference | - | Reference |  |
|  | 2-3 | 4.353 (0.569-33.280) | 0.156 | 5.361 (0.484-59.353) | 0.171 | 2.271 (0.786-6.562) | 0.130 | 1.866 (0.602-5.784) | 0.280 |
|  | 4-5 | 9.800 (1.219-78.780) | **0.032** | 3.513 (0.293-42.176) | 0.322 | 3.680 (1.169-11.587) | **0.026** | 2.082 (0.54-7.993) | 0.285 |
| MSKCC | Low | Reference | - |  |  | Reference | - |  |  |
|  | Intermediate | 1.175 (0.143-9.628) | 0.145 |  |  | 0.671 (0.228-1.980) | 0.470 |  |  |
|  | High | 4.549 (0.593-34.900) | 0.881 |  |  | 1.150 (0.386-3.428) | 0.802 |  |  |
| Path-score | Low | Reference | **<0.001** | Reference | **<0.001** | Reference | **0.006** | Reference | **0.022** |
|  | High | 5.839 (2.359-14.460) |  | 7.423 (2.738-20.119) |  | 2.405 (1.286-4.500) |  | 2.143 (1.116-4.113) |  |

**Supplementary Table S9. Univariate and multivariate Cox regression analyses of the Path-score and clinicopathological characteristics for overall**

**survival and progression-free survival in the validation cohort**

| Characteristics | Category | Overall survival  Univariate analysis Multivariate analysis  HR (95% CI) p-value HR (95% CI) p-value | | | | Progression-free survival  Univariate analysis Multivariate analysis  HR (95% CI) p-value HR (95% CI) p-value | | | |
| --- | --- | --- | --- | --- | --- | --- | --- | --- | --- |
| Age | <65 | Reference | **0.026** | Reference | **0.048** | Reference | 0.422 |  |  |
|  | >=65 | 2.984 (1.141-7.807) |  | 3.428 (1.011-11.625) |  | 1.396 (0.618-3.151) |  |  |  |
| Sex | Female | Reference | 0.063 |  |  | Reference | 0.138 |  |  |
|  | Male | 2.540 (0.951-6.784) |  |  |  | 1.798 (0.828-3.900) |  |  |  |
| Biopsy type | Surgical resection/Open biopsy | Reference | 0.538 |  |  | Reference | 0.983 |  |  |
|  | Stereotactic biopsy | 1.487 (0.420-5.262) |  |  |  | 1.011 (0.379-2.696) |  |  |  |
| Hans | GCB | Reference | 0.124 |  |  | Reference | 0.772 |  |  |
|  | Non-GCB | 0.465 (0.175-1.233) |  |  |  | 0.880 (0.370-2.094) |  |  |  |
| Tumor Size | <5cm | Reference | 0.957 |  |  | Reference | 0.541 |  |  |
|  | >=5cm | 1.028 (0.379-2.792) |  |  |  | 1.300 (0.561-3.009) |  |  |  |
| Deep Lesions | Absent | Reference | 0.732 |  |  | Reference | 0.801 |  |  |
|  | Present | 0.833 (0.292-2.372) |  |  |  | 1.125 (0.449-2.822) |  |  |  |
| Number of lesions | Single | Reference | 0.422 |  |  | Reference | 0.723 |  |  |
|  | Multiple | 1.534 (0.540-4.358) |  |  |  | 1.158 (0.515-2.602) |  |  |  |
| KPS | <70 | Reference | **0.002** | Reference | 0.073 | Reference | **0.008** | Reference | **0.022** |
|  | >=70 | 0.195 (0.070-0.545) |  | 0.292 (0.076-1.121) |  | 0.301 (0.124-0.730) |  | 0.348 (0.142-0.857) |  |
| ECOG | <3 | Reference | 0.083 |  |  | Reference | 0.325 |  |  |
|  | >=3 | 2.439 (0.889-6.694) |  |  |  | 1.495 (0.671-3.333) |  |  |  |
| IELSG | 0-1 | Reference | - | Reference | - | Reference | - |  |  |
|  | 2-3 | 1.367 (0.298-6.262) | 0.687 | 1.252 (0.228-6.864) | 0.796 | 1.389 (0.469-4.110) | 0.553 |  |  |
|  | 4-5 | 8.363 (1.317-53.096) | **0.024** | 1.201 (0.096-15.001) | 0.887 | 3.705 (0.821-16.730) | 0.089 |  |  |
| MSKCC | Low | Reference | - |  |  | Reference | - |  |  |
|  | Intermediate | 0.557 (0.150-2.067) | 0.382 |  |  | 0.646 (0.214-1.951) | 0.438 |  |  |
|  | High | 2.472 (0.578-10.581) | 0.223 |  |  | 1.909 (0.536-6.804) | 0.319 |  |  |
| Path-score | Low | Reference | **0.022** | Reference | **0.017** | Reference | **0.002** | Reference | **0.005** |
|  | High | 3.039 (1.177-7.844) |  | 4.204 (1.299-13.601) |  | 3.508 (1.574-7.817) |  | 3.243 (1.440-7.301) |  |

**Supplementary Table S10. C-index comparison of the pathomics nomogram and the Path-score model**

| Model | Training cohort  C-index (95%CI) p-value | | Validation cohort  C-index (95%CI) p-value | |
| --- | --- | --- | --- | --- |
| Pathomics nomogram | 0.849 (0.790-0.908) | **0.001** | 0.747 (0.608-0.886) | **0.009** |
| Path-score | 0.745 (0.639-0.851) |  | 0.623 (0.472-0.774) |  |

**Supplementary Table S11. AUC comparison of the pathomics nomogram and the Path-score model, related to Fig.5**

| Model | Training cohort  AUROC (95%CI) p-value | | Validation cohort  AUROC (95%CI) p-value | |
| --- | --- | --- | --- | --- |
| AUC at 1-year | | | | |
| Pathomics nomogram | 0.862 (0.772-0.953) | **0.017** | 0.802 (0.624-0.980) | 0.068 |
| Path-score | 0.785 (0.668-0.902) |  | 0.649 (0.443-0.855) |  |
| AUC at 2-year | | | | |
| Pathomics nomogram | 0.932 (0.835-1.000) | **0.040** | 0.768 (0.576-0.960) | 0.425 |
| Path-score | 0.869 (0.730-1.000) |  | 0.679 (0.445-0.913) |  |
| AUC at 3-year | | | | |
| Pathomics nomogram | 0.927 (0.787-1.000) | 0.493 | 0.938 (0.837-1.000) | **0.033** |
| Path-score | 0.973 (0.927-1.000) |  | 0.733 (0.506-0.959) |  |

**Supplementary Table S12. AUC comparison of the pathomics nomogram and the IELSG model, related to Fig.5**

| Model | Training cohort  AUROC (95%CI) p-value | | Validation cohort  AUROC (95%CI) p-value | |
| --- | --- | --- | --- | --- |
| AUC at 1-year | | | | |
| Pathomics nomogram | 0.862 (0.772-0.953) | **0.046** | 0.802 (0.624-0.980) | 0.149 |
| IELSG | 0.620 (0.494-0.747) |  | 0.662 (0.533-0.791) |  |
| AUC at 2-year | | | | |
| Pathomics nomogram | 0.932 (0.835-1.000) | **<0.001** | 0.768 (0.576-0.960) | **0.015** |
| IELSG | 0.769 (0.636-0.901) |  | 0.544 (0.331-0.758) |  |
| AUC at 3-year | | | | |
| Pathomics nomogram | 0.927 (0.787-1.000) | **0.001** | 0.938 (0.837-1.000) | **<0.001** |
| IELSG | 0.733 (0.580-0.887) |  | 0.441 (0.284-0.598) |  |

**Supplementary Table S13. AUC comparison of the pathomics nomogram and the MSKCC model, related to Fig.5**

| Model | Training cohort  AUROC (95%CI) p-value | | Validation cohort  AUROC (95%CI) p-value | |
| --- | --- | --- | --- | --- |
| AUC at 1-year | | | | |
| Pathomics nomogram | 0.862 (0.772-0.953) | **<0.001** | 0.802 (0.624-0.980) | 0.076 |
| MSKCC | 0.660 (0.524-0.796) |  | 0.689 (0.499-0.879) |  |
| AUC at 2-year | | | | |
| Pathomics nomogram | 0.932 (0.835-1.000) | **0.002** | 0.768 (0.576-0.960) | **0.008** |
| MSKCC | 0.754 (0.578-0.930) |  | 0.545 (0.345-0.746) |  |
| AUC at 3-year | | | | |
| Pathomics nomogram | 0.927 (0.787-1.000) | **<0.001** | 0.938 (0.837-1.000) | **0.013** |
| MSKCC | 0.714 (0.482-0.947) |  | 0.740 (0.601-0.880) |  |

**Supplementary Table S14. Net reclassification improvement of the pathomics nomogram**

| **Model** | **NRI (95% CI)** | **p-value** | **IDI (95% CI)** | **p-value** |
| --- | --- | --- | --- | --- |
| **Nomogram vs. Path-score** | | | | |
| Training cohort | 0.469 (0.157-0.643) | 0.004 | 0.152 (0.059-0.308) | <0.001 |
| Validation cohort | 0.457 (0.082-0.767) | 0.016 | 0.229 (0.047-0.501) | 0.004 |
